# The double face of miR-320: cardiomyocytes-derived miR-320 deteriorated while fibroblasts-derived miR-320 protected against heart failure induced by transverse aortic constriction

**DOI:** 10.1038/s41392-020-00445-8

**Published:** 2021-02-18

**Authors:** Xudong Zhang, Shuai Yuan, Huaping Li, Jiabing Zhan, Feng Wang, Jiahui Fan, Xiang Nie, Yan Wang, Zheng Wen, Yanghui Chen, Chen Chen, Dao Wen Wang

**Affiliations:** Division of Cardiology, Tongji Hospital, Tongji Medical College and Hubei Key Laboratory of Genetics and Molecular Mechanisms of Cardiologic Disorders, Huazhong University of Science and Technology, Wuhan, 430030 China

**Keywords:** Cardiology, Molecular medicine

## Abstract

MicroRNAs (miRNAs) are aberrantly expressed in the pathophysiologic process of heart failure (HF). However, the functions of a certain miRNA in different cardiac cell types during HF are scarcely reported, which might be covered by the globe effects of it on the heart. In the current study, Langendorff system was applied to isolate cardiomyocytes (CMs) and cardiac fibroblasts (CFs) from transverse aortic constriction (TAC)-induced mice. Slight increase of miR-320 expression was observed in the whole heart tissue of TAC mice. Interestingly, miR-320 was significantly elevated in CMs but decreased in CFs from TAC mice at different time points. Then, recombinant adeno-associated virus 9 with cell-type-specific promoters were used to manipulate miR-320 expressions in vivo. Both in vitro and in vivo experiments showed the miR-320 overexpression in CMs exacerbated cardiac dysfunction, whereas overexpression of miR-320 in CFs alleviated cardiac fibrosis and hypertrophy. Mechanically, downstream signaling pathway analyses revealed that miR-320 might induce various effects via targeting PLEKHM3 and IFITM1 in CMs and CFs, respectively. Moreover, miR-320 mediated effects could be abolished by PLEKHM3 re-expression in CMs or IFITM1 re-expression in CFs. Interestingly, miR-320 treated CFs were able to indirectly affect CMs function, but not vice versa. Meanwhile, upstream signaling pathway analyses showed that miR-320 expression and decay rate were rigorously manipulated by Ago2, which was regulated by a cluster of cell-type-specific TFs distinctively expressed in CMs and CFs, respectively. Together, we demonstrated that miR-320 functioned differently in various cell types of the heart during the progression of HF.

## Introduction

Heart failure (HF) is a leading public health concern with a rising socioeconomic burden worldwide.^1^ HF is a complex clinical syndrome characterized by fluid retention and dyspnea that can be elicited by left or global ventricular dysfunction.^2^ Ventricular dysfunction involves multiple pathophysiological processes, including cardiomyocyte (CM) hypertrophy, cardiac fibroblast (CF) proliferation, and macrophage activation.^3,4^ Although a wide diversity of molecular targets has been identified, the morbidity and mortality of HF are still increasing. Hence, identification of novel mechanisms and targets underlying HF are of utmost importance. A previous research on myocardial cell types by fluorescence-activated cell sorting determined that the adult murine heart was consisted of 56% CMs, 27% CFs, 10% vascular smooth muscle cells, and 7% endothelial cells.^5^ Accumulating evidence suggested that CMs and cardiac non-myocytes might contribute to the development of cardiac remodeling together.^6^

MicroRNAs (miRNAs) are a class of small non-coding RNAs that regulate gene expressions by binding to the complementary target genes.^7^ Extensive evidence from recent studies suggested that miRNAs were involved in cardiac dysfunction via different signaling pathways. Trans-differentiated CFs secreted miR-146a into extracellular vesicles, which then mediated CMs contractility damage in a failing heart.^8^ CMs-enriched miR-29 could activate Wnt signaling and induce pathological heart remodeling.^9^ In transverse aortic constriction- (TAC) induced HF mice, deficiency of miR-33 in CFs ameliorated fibrosis; however, global knockout of miR-33 deteriorated cardiac function.^10^ Silencing miR-92a-3p by locked nucleic acid-based anti-miR led to the dysregulation of autophagy-related genes in cardiac endothelial cells (ECs) and the activation of metabolism-related genes in CMs in myocardial infarction mice.^11^

In our previous study, we found that circulating miRNA-320 was increased in patients with coronary artery disease, and miR-320a could target the serum response factor, leading to atherogenesis.^12^ Furthermore, our group revealed that miR-320 mediated doxorubicin-induced cardiotoxicity by targeting vascular endothelial growth factor (VEGF) signaling pathway.^13^ Subsequently, we illustrated that miR-320 induced diabetic nephropathy via the inhibiting of MafB.^14^ Similarly, other groups found that cardiac-specific overexpression of miR-320 could enlarge the infarct size in the heart of ischemia/reperfusion mice by inhibiting heat-shock protein 20.^15^ Moreover, miR-320 was capable of mediating angiogenesis in ECs via CMs-derived exosomes.^16^ Recently, we identified that nuclear miR-320 mediated diabetes-induced cardiac dysfunction by activating the transcription of fatty acid metabolic genes to cause lipotoxicity in the heart.^17,18^ These data indicate that miR-320 plays vital roles in cardiovascular diseases.

RNA-sequencing and microarray technology are usually applied to screen the differentially expressed miRNAs in diseases. An RNA-sequencing study that utilized the myocardium tissues and the plasma from HF patients discovered a series of miRNAs with altered expression, among which miR-320 did not appear in the top fold-change list.^19^ However, the mild difference in miR-320 levels still showed statistical significance according to the raw data (*p* = 0.007 in HF myocardium tissues and *p* = 0.004 in HF plasma, respectively).^19^ Similarly, according to a miRNA-sequencing research, the expression of miR-320 in the cardiac tissue increased slightly after TAC operation based on the raw data, although it did not show any significant difference between TAC and control groups.^20^ High-throughput sequencing (HTS) is a widely accepted technique to map the entire transcriptome in a relatively unbiased way. Due to the limitation of length, HTS-based miRNA expression data might not represent its actual abundance, and quantitative real-time PCR is usually performed to validate the specific miRNAs screened out by HTS. However, negative data from HTS seldom attract attentions. Although the changes in the expression of miR-320 are not obvious in HF patients, miR-320 fulfills critical functions in cardiovascular diseases. Therefore, the effects of miR-320 on HF progression should be investigated.

In the current study, we investigated the miR-320 expression pattern to determine whether miR-320 was differently changed in specific cell types of the heart and the roles of miR-320 in HF.

## Results

### MiR-320 expression was increased in HF and its expression responded differently to Angiotensin II in primary CMs and CFs

**Fig. 1:**
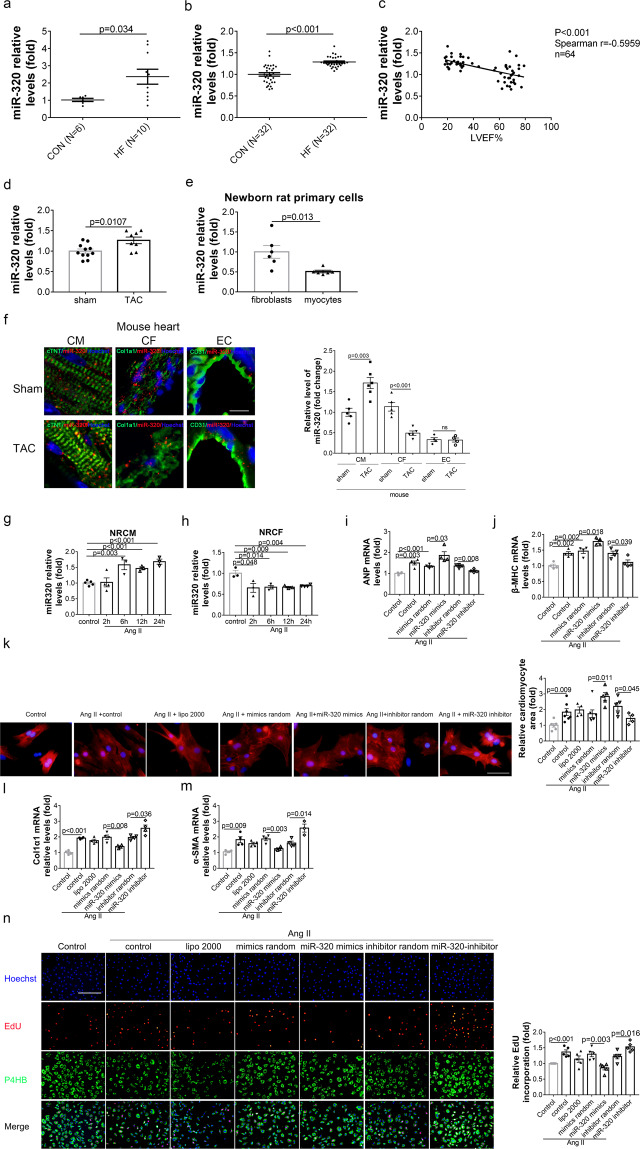
MiR-320 expression was increased in HF and its expression responded differently to Ang II in primary CMs and CFs. **a** Real-time PCR analysis of miR-320 expression in human myocardial tissues. **b** Real-time PCR analysis of miR-320 expression in human plasma. **c** Correlation analysis between miR-320 levels in plasma and LVEF%. **d** The levels of miR-320 in sham mouse hearts (*n* = 11) and TAC-induced mouse hearts (*n* = 8). **e** The relative expressions of miR-320 in NRCFs and NRCMs (*n* = 6 per group). **f** Colocalization of miR-320 with the myocyte marker cTNT, fibroblast marker col1α1 and endothelial marker CD31 in control and HF heart sections of and mouse detected by fluorescent in situ hybridization (left), quantified by Image J (right). **g** Real-time PCR analysis of miR-320 by Ang II stimulating at different time points in NRCMs (*n* ≥ 3). **h** Real-time PCR analysis of miR-320 by Ang II stimulating at different time points in NRCFs (*n* ≥ 3). **i** Expression of ANP mRNA in NRCMs with different treatments measured by real-time PCR (*n* ≥ 3). **j** Expression of β-MHC mRNA in NRCMs with different treatments measured by real-time PCR (*n* ≥ 3). **k** Representative images of CMs areas stained by ACTN2 (left) and the quantitative analysis of cell sizes (right). Scale bar, 100 µm. **l** Col1a1 mRNA levels of NRCFs with different treatments detected by real-time PCR (*n* ≥ 3). **m** Expression of α-SMA mRNA in NRCFs with different treatments measured by real-time PCR (*n* ≥ 3). **n** Representative images of immunofluorescence staining for EdU (red), Hoechst (blue) and P4HB (green) in NRCFs with different treatments (left). Scale bar, 100 µm. Quantitative analysis of EdU measured by Image J (right). NRCFs newborn rat cardiac fibroblasts, NRCMs newborn rat cardiomyocytes, TAC transverse aortic constriction, Ang II Angiotensin II, cTNT cardiac muscle troponin T; Data are presented as mean ± SEM

Quantitative RT-PCR assays showed that miR-320 was slightly elevated in the heart tissues and the plasma from HF patients (Fig. 1a, b and Supplementary Tables 1 and 2). Meanwhile, the expression of circulating miR-320 was negatively correlated with the left ventricular ejection fraction (LVEF; Fig. 1c). In line with this, miR-320 expression was slightly increased in the global heart tissues from TAC mice at 6 weeks as compared with the sham mice (Fig. 1d).

Simultaneously, miR-320 was abundant in both primary CMs and CFs isolated from normal rat heart (Fig. 1e). Moreover, fluorescence in situ hybridization (FISH) analysis showed that miR-320 was presented in CMs (cTNT positive cells) and its expression in CMs was significantly increased in TAC-induced HF mice (Fig. 1f). In contrast, although miR-320 signal was also co-localized with fibroblast- and endothelial-specific markers, its expression was decreased in CFs while remained unchanged in ECs during HF (Fig. 1f). Because the protein level of Angiotensin II (Ang II) was increased in the heart of TAC-treated mice (Supplementary Fig. 1a) and Ang II was commonly used to induce CMs hypertrophy and CFs proliferation in vitro,^21,22^ isolated primary CMs and CFs were then treated with Ang II in vitro. Interestingly, Ang II treatment increased miR-320 expression at 6 h, and then miR-320 expression remained at a relatively high level (1.5-fold relative to control) until 24 h in neonate rat cardiomyocytes (NRCMs; Fig. 1g). Conversely, in neonate rat cardiac fibroblasts (NRCFs), miR-320 expression declined at 2 h after Ang II treatment and maintained at a fairly low level (0.7-fold relative to control) until 24 h (Fig. 1h). The purity of NRCMs and NRCFs was confirmed by immunofluorescence assays (Supplementary Fig. 2a, b).

In NRCMs, ANP and β-MHC expressions, as well as CM area, showed no significant change due to miR-320 treatment under normal condition (Supplementary Fig. 3a–d). However, overexpression of miR-320 enhanced the Ang II-induced increase of ANP and β-MHC mRNA levels, whereas inhibition of miR-320 showed contrasting effects (Fig. 1i, j). Morphology analysis indicated that miR-320 could promote Ang II-induced hypertrophy (Fig. 1k).

In NRCFs, the elevated mRNA levels of the fibrosis markers, col1α1 and α-SMA, caused by Ang II treatment were decreased by the transfection with miR-320 (Fig. 1l, m). Moreover, EdU assays showed that overexpression of miR-320 could restrain cell proliferation, while inhibition of miR-320 could promote cell proliferation under stress conditions (Fig. 1n). Under normal conditions, although no significant changes in col1α1 and α-SMA expressions were introduced by miR-320 treatment, overexpression of miR-320 inhibited cell proliferation without Ang II treatment (Supplementary Fig. 3e–h).

Hence, the miR-320 expression level was mildly enhanced in the global heart of HF, while the reverse expression patterns were observed in different cell types. Moreover, miR-320 functioned differently in primary CMs and CFs in vitro.

### MiR-320 expression patterns were opposite between isolated CMs and CFs from TAC mice

**Fig. 2:**
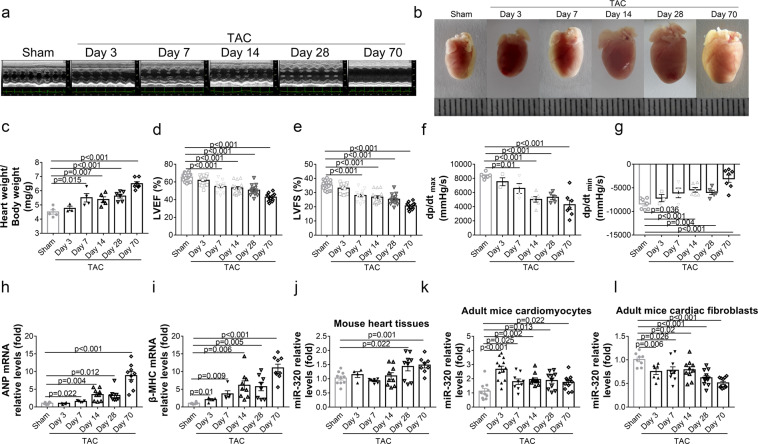
MiR-320 expression patterns were opposite between isolated CMs and CFs from TAC mice. **a** C57BL/6 mice were killed to either sham operation (*n* = 16) or TAC and noted after 3, 7, 14, 28, or 70 days (*n* = 15, 15, 17, 14, or 13, respectively). Representative short axis images of echocardiograms. **b** Gross morphologies of hearts from mice after TAC at different time points are shown. **c** The ratios of heart weight and body weight in mice after TAC. **d**, **e** LVEF% and LVFS% were detected by echocardiography. **f**, **g** Hemodynamic parameters of mice with different treatments were measured by the Millar cardiac catheter system. dp/dt_max_, peak instantaneous rate of LV pressure increase; dp/dt_min_, peak instantaneous rate of LV pressure decrease. **h**, **i** ANP and β-MHC mRNA levels in the hearts were detected by real-time PCR. **j** MiR-320 expression levels in the hearts were quantified by real-time PCR. **k** The expressions of miR-320 in isolated CMs were measured by real-time PCR. **l** Real-time PCR analysis of miR-320 in isolated CFs. ANP Atrial natriuretic peptide, β-MHC β-myosin heavy chain, LVEF left ventricular ejection fraction, LVFS left ventricular fractional shortening; Data are presented as mean ± SEM

To further investigate the expression patterns of miR-320 in different cell types of the failing hearts, wild type C57BL/6 mice subjected to TAC were killed at multiple time points. The echocardiographic analyses showed that the blood velocity in the aortic arch was sharply increased in mice receiving TAC surgery (Supplementary Fig. 4a, b). Moreover, short-axis images suggested that the left ventricular chamber was gradually enlarged (Fig. 2a). Consistently, the TAC mice exhibited augmented heart size (Fig. 2b) and heart weight to body weight (HW/BW) radio (Fig. 2c), but reduced LVEF (Fig. 2d) and left ventricular fractional shortening (LVFS) (Fig. 2e) beginning on 7 day after TAC (Supplementary Table 3). Hemodynamics analysis found similar changes, indicated by gradually decreased dp/dt_max_ and dp/dt_min_ (Fig. 2f, g). Consistently, the expression levels of HF biomarkers, ANP and β-MHC, were elevated in the heart after TAC (Fig. 2h, i).

Most important, the miR-320 expressions in the global heart tissues were only increased beginning on 28 day after TAC, which was the advanced HF stage (Fig. 2j). Meanwhile, we isolated the primary CMs and CFs at different time points after TAC, respectively (Supplementary Fig. 5a–c). Interestingly, we found that miR-320 expressions in CMs were rapidly reached its peak 3 day after TAC, and then remained at an elevated level until 70 day (Fig. 2k). Conversely, in CFs, miR-320 expressions reduced sharply 3 day after TAC, and then continued to decline until 70 day (Fig. 2l).

Our data showed that although the overall change was not obvious, the changes of miR-320 in CMs and CFs were significant and different after TAC.

### Overexpression of miR-320 in CMs aggravated HF in vivo

**Fig. 3:**
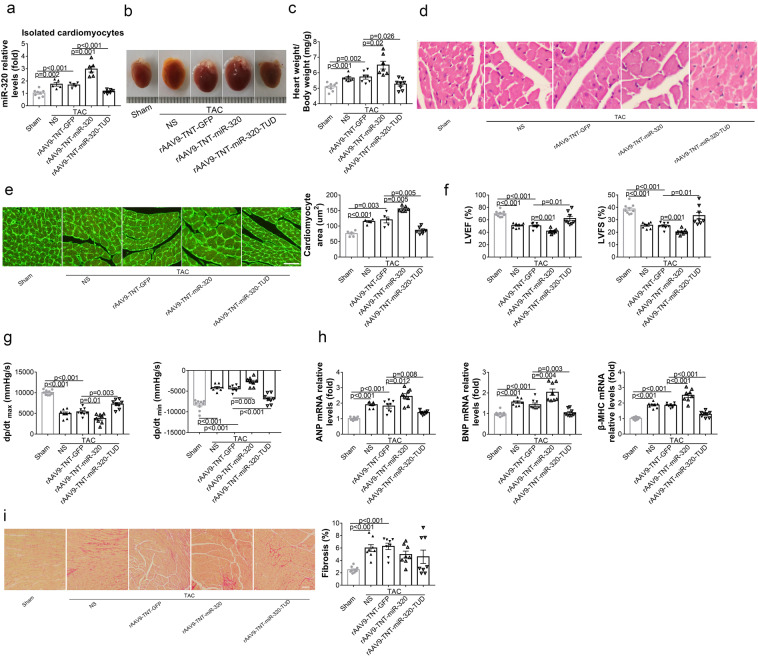
Overexpression of miR-320 in CMs aggravated HF in vivo. **a** Relative miR-320 expression in isolated CMs measured by real-time PCR. **b** Representative gross morphologies of hearts from mice subjected to different treatments. **c** The ratios of heart weight to body weight in mice with diverse treatments. **d** Representative images of transverse area of CMs detected by H&E. Scale bars, 50 µm. **e** Histological analysis of transverse area of CMs measured by WGA staining (left). Scale bars, 25 µm. The areas of CMs were analyzed by Image-Pro Plus (right). **f** Echocardiography analysis of LVEF%, LVFS%. **g** Hemodynamic parameters (dp/dt_max_ and dp/dt_min_) were measured by the Millar cardiac catheter system. **h** Relative mRNA expressions of cardiac hypertrophy markers in heart tissues from treated mice. **i** Representative images of Sirius Red staining of heart sections from mice with different treatments (left), and the quantification analysis of cardiac fibrosis (right). Scale bars, 50 µm. H&E hematoxylin and eosin, WGA wheat germ agglutinin. Sham (*n* = 9), TAC + NS (*n* = 8), TAC + rAAV9-TNT-GFP (*n* = 8), TAC + rAAV9-TNT-miR-320 (*n* = 8), TAC + rAAV9-TNT-miR-320-TUD (*n* = 8). Data are expressed as mean ± SEM

To explore the direct effects of miR-320 on CMs in vivo, rAAV9-TNT-miR-320 was employed in TAC mice to modulate the expressions of mature miR-320 in CMs specifically (Supplementary Fig. 6a). As detected by quantitative RT-PCR, miR-320 expression was increased in the isolated CMs from TAC mice. Moreover, rAAV9-TNT-miR-320 treatment increased miR-320 expression, while rAAV9-TNT-miR-320-TUD delivery reduced the expression of miR-320 in isolated CMs from TAC mice (Fig. 3a). TAC-induced increases in heart size and the HW/BW ratio were further aggravated by the overexpression of miR-320 in CMs, whereas the inhibition of miR-320 showed the opposite effects (Fig. 3b, c). Moreover, CM morphology measured by hematoxylin and eosin (HE) and wheat germ agglutinin (WGA) staining confirmed the pro-hypertrophy effects of miR-320 (Fig. 3d, e). The echocardiographic analysis suggested that upregulated miR-320 in CMs further deteriorated the cardiac function in TAC mice, whereas downregulated miR-320 in CMs improved the cardiac function (Fig. 3f). Hemodynamics analysis by Millar catheter showed similar changes (Fig. 3g). Meanwhile, the elevated expressions of ANP, BNP, and β-MHC in TAC mice were enhanced by CM-specific miR-320 overexpression, but reduced by CM-specific miR-320 inhibition (Fig. 3h). However, Sirius Red staining showed that TAC-induced myocardial fibrosis was not affected by the injection of rAAV9-TNT-miR-320 or rAAV9-TNT-miR-320-TUD (Fig. 3i), which suggested that CM-specific expression of miR-320 might not impact the function of CFs.

These data indicated that CM-specific enhanced miR-320 expression could worsen cardiac hypertrophy in TAC-induced HF mice without affecting the function of CFs.

### Overexpression of miR-320 in CFs mitigated HF in vivo

**Fig. 4:**
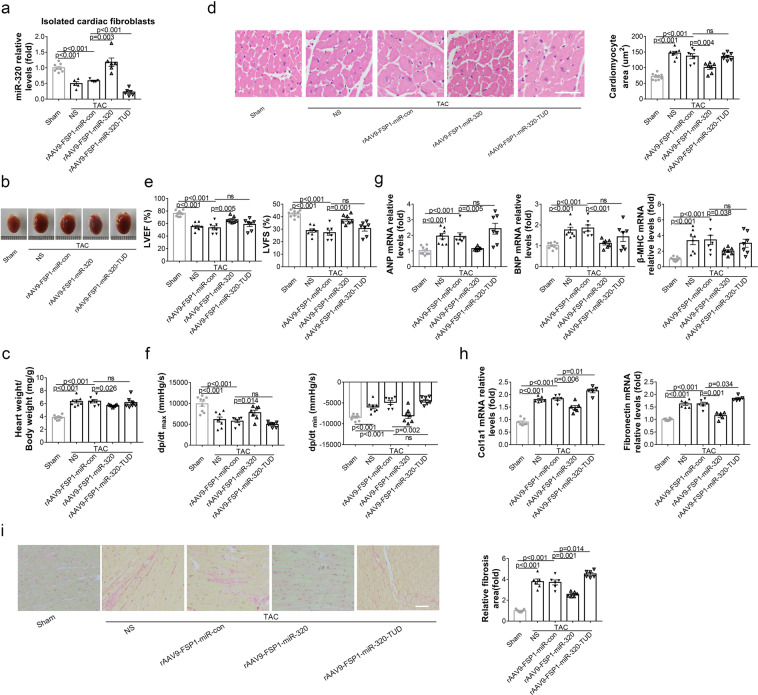
Overexpression of miR-320 in CFs mitigated HF in vivo. **a** Relative expression of miR-320 in isolated CFs detected by real-time PCR. **b** Representative gross morphologies of the hearts in mice subjected to different treatments. **c** The ratios of heart weight to body weight in mice with diverse treatments. **d** H&E staining represented the areas of CMs (left). Scale bars, 50 µm. The areas of CMs were analyzed by Image-Pro Plus (right). **e** Echocardiography analysis of LVEF% and LVFS%. **f** Hemodynamic parameters were measured by the Millar cardiac catheter system. **g** Relative mRNA expressions of cardiac hypertrophy markers in the heart tissues from diversely treated mice. **h** Expression levels of markers of cardiac fibrosis in the heart from mice with different treatments measured by real-time PCR. **i** Representative images of Sirius Red staining of the heart sections from mice with different treatments (left) and the quantification analysis of cardiac fibrosis (right). Scale bars, 50 µm. Sham (*n* = 9), TAC + NS (*n* = 8), TAC + rAAV9-FSP1-miR-con (*n* = 7), TAC + rAAV9-FSP1-miR-320 (*n* = 7), TAC + rAAV9-FSP1-miR-320-TUD (*n* = 7). Data are expressed as mean ± SEM

Meanwhile, TAC mice were treated with rAAV9-FSP1-miR-320 or rAAV9-FSP1-miR-320-TUD, respectively, to manipulate the expression of miR-320 in CFs specifically (Supplementary Fig. 6b). As shown in Fig. 4a, miR-320 expression was decreased in the isolated CFs from TAC mice. Furthermore, rAAV9-FSP1-miR-320 delivery enhanced the miR-320 levels, whereas rAAV9-FSP1-miR-320-TUD inhibited the expression of miR-320 in isolated CFs of TAC mice. Contrary to the effects of CM-specific miR-320, overexpression of miR-320 in CFs ameliorated the increased heart size and HW/BW ratio in TAC mice (Fig. 4b, c). In addition, the enlarged CM area in TAC mice was decreased by CF-specific overexpression of miR-320 (Fig. 4d).

Further, echocardiographic analysis and hemodynamic analysis exhibited improved cardiac function by CF-specific overexpression of miR-320 (Fig. 4e, f). Moreover, the effects of CF-specific miR-320 on the mRNA levels of ANP, BNP, and β-MHC were improved, which were in line with the cardiac function (Fig. 4g). Meanwhile, mRNA levels of col1α1 and fibronectin showed that overexpression of miR-320 in CFs inhibited TAC-induced fibrosis, while inhibition of miR-320 in CFs increased fibrosis (Fig. 4h). Sirius Red staining presented consistent results with the mRNA levels of fibrosis markers (Fig. 4i). Remarkably, although the downregulation of miR-320 in CFs by rAAV9-FSP1-miR-320-TUD could slightly increase fibrosis, it could not aggravate cardiac hypertrophy and dysfunction in TAC mice (Fig. 4).

Hence, overexpression of miR-320 in CFs could attenuate TAC-induced HF. Despite a slight increase in fibrosis, further downregulation of miR-320 in CFs did not exacerbate the impaired cardiac function in TAC mice (See further in Discussion section).

Notably, at baseline, no significant difference was observed among these mice with different treatments (Supplementary Fig. 6c–e), indicating that miR-320 selectively affected cardiac function under stress conditions (See further in Discussion section).

### The different expression patterns of miR-320 were governed by argonaute2

**Fig. 5:**
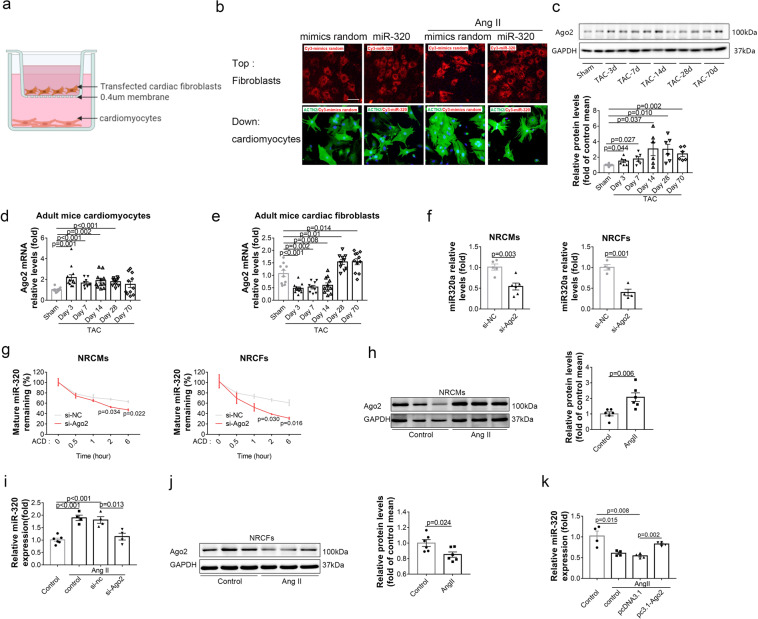
The different expression patterns of miR-320 were governed by Ago2. **a** Schematic diagram of co-culture assay. Top well, NRCFs; lower chamber, NRCMs. **b** NRCFs in the top chamber were photographed by fluorescence microscope after transfected with Cy3-labeled miR-320. ACTN2 staining of NRCMs in the lower chamber. Scale bar, 100 µm. **c** Protein expression levels of Ago2 and GAPDH in mouse hearts after TAC (left). Quantitative analysis of Ago2 protein levels after normalization to GAPDH (right). **d** Ago2 mRNA levels in CMs at different time points after TAC were measured by real-time PCR. Sham (*n* = 10), TAC-Day 3 (n = 13), TAC-Day 7 (*n* = 9), TAC-Day 14 (*n* = 11), TAC-Day 28 (*n* = 10), and TAC-Day 70 (*n* = 11). **e** Ago2 mRNA levels in CFs at different time points after TAC were measured by real-time PCR. Sham (*n* = 10), TAC-Day 3 (*n* = 12), TAC-Day 7 (*n* = 9), TAC-Day 14 (*n* = 11), TAC-Day 28 (*n* = 10), and TAC-Day 70 (*n* = 11). **f** Relative miR-320 levels in si-Ago2 treated NRCMs (left, *n* ≥ 3) and NRCFs (right, *n* ≥ 3). **g** Mature miR-320 levels detected by real-time PCR after actinomycin D treatment, normalization to GAPDH in NRCMs (left, *n* = 4) and NRCFs (right, *n* = 4). **h** Protein expression levels of Ago2 and GAPDH in NRCMs with Ang II treatment (left). Quantitative analysis of Ago2 protein levels after normalization to GAPDH (right). **i** MiR-320 expression levels in NRCMs with different treatments were measured by real-time PCR (*n* ≥ 3). **j** Protein expression levels of Ago2 and GAPDH in NRCFs with Ang II treatment (left). Quantitative analysis of Ago2 protein levels after normalization to GAPDH (right). **k** MiR-320 expression levels in NRCFs with different treatment were measured by real-time PCR (*n* ≥ 3). Data are expressed as mean ± SEM

The in vivo study revealed that CF-specific miR-320 overexpression protected against TAC-induced CMs hypertrophy (Fig. 4d), indicating a potential cell-cell crosstalk between CFs and CMs. To determine whether CFs treated with miR-320 could affect CMs hypertrophy and the underlying mechanism, transwell co-culture assays were performed. Firstly, CFs were transfected with Cy3-labeled miR-320 and then laid on the top well of the system. Meanwhile, CMs were grown in the bottom well (Fig. 5a). After co-culture, we noted that miR-320 was only detectable in CFs but not in CMs (Fig. 5b), indicating that miR-320 transfected into CFs was unable to further translocate into CMs. Strikingly, cardiac hypertrophy markers were significantly decreased in CMs co-cultured with miR-320 transfected CFs compared with miR-control transfected CFs under Ang II stress (Supplementary Fig. 7a). These data suggested that miR-320 treated CFs were able to affect the expression of hypertrophy markers in CMs, but miR-320 itself was unable to transfer from CFs into CMs. Then, we performed LC-MS proteomics on the cell supernatant to identify the potential signals mediating the crosstalk between CFs and CMs. Interestingly, a cluster of proteins altered in the Ang II-treated supernatant were rescued by miR-320 transfection in CFs, which might regulate the expression of hypertrophy markers in CMs (Supplementary Fig. 7b).

Conversely, CMs were transfected with Cy3-labeled miR-320 and then laid on the top well of the transwell system with CFs grown in the bottom well (Supplementary Fig. 7c). Similarly, miR-320 was only detectable in CMs but not in CFs, indicating that miR-320 transfected into CMs was unable to translocate into CFs neither (Supplementary Fig. 7d). Interestingly, proliferation was unchanged in CFs co-cultured with miR-320 transfected CMs compared with miR-control transfected CMs under Ang II stress (Supplementary Fig. 7e). Moreover, the transwell experiments were repeated in mouse fibroblast NIH3T3 cell line and cardiomyocyte HL-1 cell line (Supplementary Fig. 7f, g). The consistent results observed in rat and mouse cells strongly supported the conserved function of miR-320 among species.

These in vitro data illustrated the complex crosstalk between CFs and CMs that miR-320 treated CFs were able to indirectly affect CMs function, but not vice versa.

We then determined to investigate the mechanism that governed the distinct expression patterns of miR-320 in CMs and CFs, respectively. Recently, some research groups reported that argonaute2 (Ago2) mediated the biogenesis of erythroid miRNAs.^23,24^ Moreover, the methylation of Ago2 mRNA could repress miRNA expression in the process of human aging.^25^ Hence, we measured the protein levels of Ago2 in the total heart tissues of TAC mice at multiple time-points. Consistent with the expression patterns of miR-320, Western blot assays indicated that Ago2 protein levels increased by 3 day, peaked at the 14 day, and maintained at a high level until 70 day after TAC (Fig. 5c). In isolated primary CMs, mRNA levels of Ago2 reached its peak at 3 day and maintained a high level until 70 day after TAC (Fig. 5d). While in isolated primary CFs, comparing to the sham group, Ago2 mRNA expression decreased continuously until 14 day after TAC, but increased beginning on 28 day after TAC (Fig. 5e). These data indicated that at the early time points after TAC, the Ago2 mRNA expression patterns were coordinated with the miR-320 expression patterns in CMs and CFs, respectively.

Then, small interfering RNA (siRNA) was applied to downregulate the Ago2 expression in NRCFs and NRCMs, respectively (Supplementary Fig. 8a, b). The mature miR-320 expressions were decreased in both NRCFs and NRCMs treated with si-Ago2 (Fig. 5f). Strikingly, the miR-320 decay rate was increased in NRCMs and NRCFs treated with Ago2 siRNAs, indicating the compromised miR-320 stability upon Ago2 loss (Fig. 5g). Furthermore, to rule out the potential transcriptional regulation of Ago2 on miR-320 expression, we evaluated the primary miR-320 levels, finding that pri-miR-320 remained unchanged by Ago2 knockdown (Supplementary Fig. 8c). Moreover, luciferase assays revealed unchanged fluorescence signal of pGL3-miR-320 promotor by Ago2 overexpression (Supplementary Fig. 8d). These data argued against the direct regulation of Ago2 on miR-320 transcription. Moreover, the protein expressions of Ago2 were increased after Ang II treatment in NRCMs (Fig. 5h). Most importantly, the downregulation of Ago2 in NRCMs reduced Ang II-induced miR-320 increases in NRCMs (Fig. 5i). Alternatively, Ang II treatment decreased Ago2 expressions in NRCFs (Fig. 5j). While the overexpression of Ago2 enhanced Ang II-induced miR-320 decrease in NRCFs (Fig. 5k).

Further, we explored what might contribute to the different regulation patterns of Ago2 under Ang II stress in CMs versus CFs. Because mRNA levels of Ago2 were also changed, we suspected that some cell-type-specific transcriptional factors (TFs) might be responsible for the re-arranged Ago2 expression. We screened the potential TFs which could directly regulate Ago2 transcription using JASPAR, a database of TFs binding profiles. Results showed that 15 TFs might target the promoter region of Ago2 in human, mouse and rat, conservatively (Supplementary Table 4). Meanwhile, five TFs (ELF1, KLF4, STAT1, STAT5, and ZFX) were abundant in CMs and CFs (FPKM ≥ 0.1), according to a single-cell sequencing study on healthy adult mice in primary CMs and CFs.^26^ Notably, among these TFs, ELF1 and KLF4 were significantly increased after Ang II treatment in NRCMs, while STAT1 levels were decreased in Ang II-treated NRCFs (Supplementary Fig. 8e, f). Interestingly, we noted that ELF1 was abundant in CMs, while STAT1 was more enriched in CFs (Supplementary Fig. 8g, h). Moreover, luciferase assays revealed a direct activation of these two TFs on Ago2 transcription (Supplementary Fig. 8i). Therefore, the different expression patterns of Ago2 under Ang II treatment seemed to be mediated by a cluster of cell-type-specific TFs distinctively expressed in CMs and CFs, respectively.

These data suggested that the different expression patterns of miR-320 in CMs and CFs might be governed by Ago2, rather than the interaction between these two cell types upon stress signals.

### miR-320 targeted different signals in CMs and CFs

**Fig. 6:**
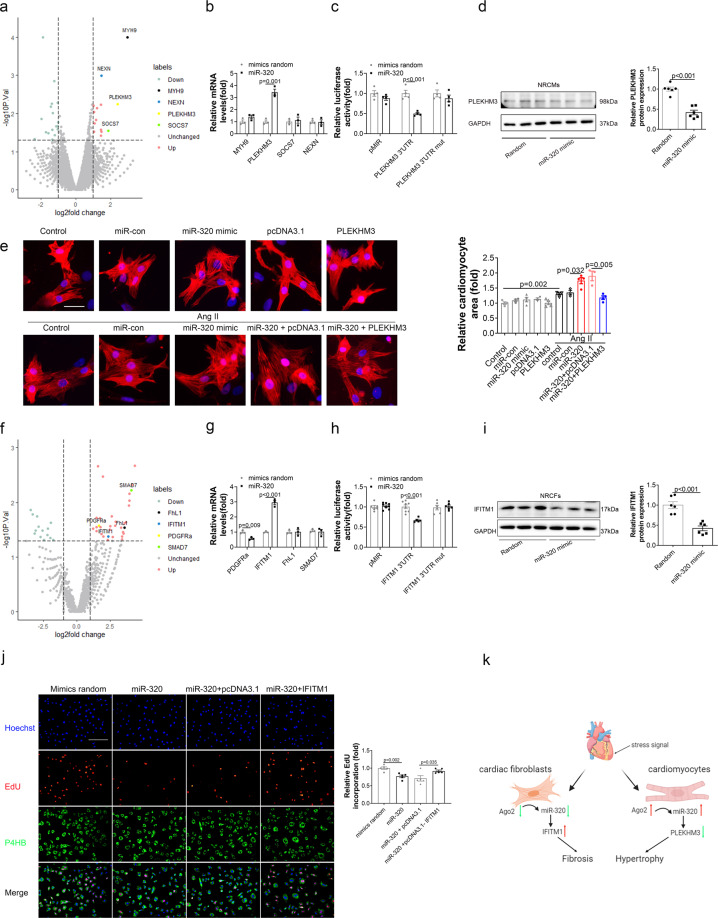
MiR-320 targeted different signals in CMs and CFs. **a** Volcano plot of RIP-seq in H9c2 cells. **b** The levels of mRNA in miR-320-transfected NRCMs detected by RNA binding protein immunoprecipitation (*n* ≥ 3). **c** Regulation of miR-320 on the 3′-UTR of plekhm3 detected by luciferase reporter assays in HEK293 cells. **d** The protein levels of plekhm3 in NRCMs with different treatments were detected by western blot (left), quantified by Image J (right; *n* = 6). **e** Representative images of CMs areas stained by ACTN2 (left), and quantitative analysis of cell sizes (right). Scale bar, 100 µm. **f** Volcano plot of RIP-seq in NRCFs. **g** The levels of mRNA in miR-320-transfected NRCFs detected by RNA binding protein immunoprecipitation (*n* ≥ 3). **h** Regulation of miR-320 on the 3′-UTR of IFITM1 detected by luciferase reporter assays in HEK293T cells (*n* ≥ 3). **i** The protein level of IFITM1 in NRCFs with different treatments detected by western blot (left), quantified by Image J (right; *n* = 6). **j** Representative images of immunofluorescence staining for EdU (red), Hoechst (blue), and P4HB (green) in NRCFs with different treatments (left). Scale bar, 100 µm. Quantitative analysis of EdU measured by Image J (right). **k** A model to illustrate the roles of miR-320 in CFs and CMs during HF. Data are expressed as mean ± SEM

To map the downstream signals of miR-320, RIP-seq was conducted in CMs and CFs, respectively. Figure 6a showed the volcano plot of the RIP-seq in CMs. Genes with the upregulated combination were listed in Supplementary Table 5. Among these upregulated genes, myosin heavy chain 9 (myh9), pleckstrin homology domain containing M3 (plekhm3), suppressor of cytokine signaling 7 (socs7), and nexilin filamentous-actin binding protein (nexn) possessed potential binding sites with miR-320 in human, rat and mouse according to RNAhybrid software. However, only plekhm3 was further confirmed by RIP-PCR in NRCMs (Fig. 6b). Then luciferase assay indicated that plekhm3 3′-UTR reporter treated with miR-320 was significantly suppressed compared with random mimics treatment or 3′-UTR mutant reporter (Fig. 6c). The protein level of plekhm3 in NRCMs was also reduced after transfection with miR-320 (Fig. 6d). In the heart tissues of rAAV9-TNT-miR-320 treated mice, PLEKHM3 was exclusively downregulated by miR-320 overexpression in CMs (Supplementary Fig. 9a). Meanwhile, miR-320 treated HL-1 mouse cardiac cells and AC16 human cardiac cells also showed the same results (Supplementary Fig. 9b, c).

Moreover, siRNA-plekhm3 expanded Ang II-induced increase in ANP and β-MHC expressions, while plekhm3 overexpression decreased the expression of these genes in CMs (Supplementary Fig. 9d, e). In addition, overexpression of plekhm3 reversed the miR-320 induced cell area enlargement and reduced hypertrophy related gene levels in Ang II-treated CMs (Fig. 6e and Supplementary Fig. 9f).

Meanwhile, RIP-seq was conducted to screen the potential targets of miR-320 in CFs. Figure 6f indicated the volcano plot of RIP-seq in NRCFs. The upregulated genes with statistically significance were presented in Supplementary Table 6. According to RNAhybrid software, platelet derived growth factor receptor alpha (PDGFRα), interferon induced transmembrane protein 1 (IFITM1), four and a half LIM domains 1 (FhL1), and SMAD family member 7 (SMAD7) could be targeted by miR-320 in human, rat and mouse. Among these genes, RIP-PCR in NRCFs indicated that only IFITM1 mRNA was increased with Ago2 after miR-320 transfection among these genes (Fig. 6g). Meanwhile, the results of luciferase assays suggested that the relative luciferase activity of pMIR-IFITM1 3′-UTR was significantly inhibited by miR-320 (Fig. 6h). Western blot assays also showed consistent results (Fig. 6i). IFITM1 was specifically suppressed by miR-320 overexpression in CFs (Supplementary Fig. 9g). Similar results were also observed in NIH3T3 cells (Supplementary Fig. 9h). Moreover, overexpression of IFITM1 could attenuate the effects of miR-320 on CFs (Fig. 6j and Supplementary Fig. 9i). Meanwhile, we analyzed IFITM1 and PLEKHM3 protein levels in CFs and CMs after TAC, finding that IFITM1 was increased in CFs while remained unchanged in CMs (Supplementary Fig. 9j). In contrast, PLEKHM3 was downregulated in CMs while remained unchanged in CFs (Supplementary Fig. 9k). The re-arranged expression of IFITM1 and PLEKHM3 were coordinated with miR-320 expression changes.

In summary, stress could induce Ago2 and miR-320 expressions in CMs but reduce Ago2 and miR-320 expressions in CFs. Overexpression of miR-320 in CMs exacerbated TAC-induced cardiac hypertrophy whereas miR-320 overexpression in CFs generated the opposite effects via different signals (Fig. 6k).

## Discussion

In this study, we report the different effects of miR-320 on CMs and CFs during HF, respectively. In particular, TAC-induced increase of miR-320 in CMs caused the deterioration of cardiac function, whereas the downregulated miR-320 in CFs induced cardiac fibrosis and dysfunction in TAC mice. Interestingly, the different expression patterns of miR-320 in CM and CF were governed by Ago2.

In fact, miR-320 has been wildly studied previously. However, miR-320 seems to function differently in various cell types. In CMs, overexpression of miR-320 enhanced CMs death and apoptosis, whereas knockdown of miR-320 was cytoprotective against ischemia/reperfusion stimulation.^15^ In fibroblasts, miR-320 acted in stromal fibroblasts to reprogram the tumor microenvironment and curtailed tumor progression.^27^ Our previous study revealed that miR-320 overexpression inhibited human-derived ECs proliferation, induced apoptosis and promoted atherogenesis.^12^ Interestingly, it came to our notice that another well-studied miRNA, miR-21 also exerted contradictive roles on CMs and CFs. Systemic inhibition of miR-21 has proven effective against myocardial fibrosis and dysfunction, but studies in CMs suggested a protective role of miR-21.^28–30^ Considering the potential implications of miR-320 based therapy against HF, we aimed to determine the functions of miR-320 in certain cell types during disease processes. Our data indeed revealed that miR-320 exerted opposite functional properties in CMs versus CFs and encouraged further development of miR-320 based therapy towards cellular tropism.

Interestingly, we noted that Ago2 overexpression positively regulated while Ago2 knockdown decreased miR-320 expression in CMs and CFs. In fact, this observation was in line with a previous study showing decreased hepatic miR-320 levels by liver-specific Ago2-deletion.^31^ Based on the published studies, Ago2 was able to regulate gene expressions at both translational and post-translational levels.^18,32^ By directly binding to DNA promoter region, Ago2 could directly induce transcriptional gene activation or transcriptional gene silencing, depending on the TFs recruited. At post-transcriptional levels, Ago2 could bind with small non-coding RNAs and regulate protein synthesis, affect messenger RNA stability.^33^ In terms of the regulation of Ago2 on miRNAs, a recent study demonstrated that non-canonical miRNAs were decreased upon Ago2 loss and could be rescued by expressing Ago2, confirming that Ago2 protein was required for the stability of small RNAs specifically synthesized by promoter-proximal RNA polymerase II.^34^ Very interestingly, miR-320 was one of the few miRNAs that directly synthesized by RNA polymerase II.^35^ Strikingly, miR-320 decay rate was increased in CMs and CFs treated with Ago2 siRNAs, indicating the compromised miR-320 stability upon Ago2 loss. These data argued against the direct regulation of Ago2 on miR-320 transcription. Our study revealed that Ago2 was required for the stability of mature miR-320 in CMs and CFs, which extended the understanding of miRNA biogenesis and function.

In fact, a cluster of other miRNAs might be also regulated by Ago2,^34^ therefore their potential roles in CFs and CMs could not be completely ruled out. For example, miR-29 was also synthesized by RNA polymerase II and regulated by Ago2 (Supplementary Fig. 10a, b). Therefore, it is possible that Ago2 might cause some other effects through regulating these miRNAs. A key strategy to deal with this issue is to observe the ability of an elucidated target to phenocopy the effects of Ago2. We therefore performed the target rescue experiments. Our in vitro data showed that miR-320 overexpression abolished Ago2 knockdown mediated hypertrophy and proliferation in CMs and CFs (Supplementary Fig. 11a, b), respectively, suggesting that miR-320 was a key target of Ago2 in the disease process. Whether other RNA polymerase II- and non-RNA polymerase II-derived miRNAs could be regulated by Ago2 similarly, and whether other RNA binding proteins would engage are intriguing subjects for future study.

Moreover, the different regulation patterns of Ago2 under stress seemed to be mediated by a cluster of cell-type-specific TFs distinctively expressed in CMs and CFs, respectively. In line with our study, previous study also demonstrated different regulation patterns of various TFs among CMs, CFs, ECs and macrophages during HF.^26^ While we are still pursuing to determine whether Ang II regulates these cell-type-specific TFs through transcriptional, translational or post-translational manner, the existing data provided evidence that a cluster of cell-type-specific TFs were responsible for the different destinations of Ago2 in CMs and CFs under stress.

The Ago2 mRNA expression is coordinated with miR-320 expression at the early time points after TAC (3, 7, and 14 day after TAC). However, after 28 day, the Ago2 expression level significantly increased, whereas the miR-320 expression level was still low. To address this issue, we detected the primary miR-320 levels, finding that pri-miR-320 was decreased 28 day after TAC (Supplementary Fig. 12a). We therefore suspected that at the later time points (28 day after TAC), miR-320 might be also regulated via transcriptional manner. We tested whether SP1, a TF we have previously shown directly enhanced miR-320 transcription,^12^ might be one of the contributors for the decreased miR-320 at the later time points. Interestingly, we observed a decrease of SP1 in CFs at the later time points (28 day after TAC), while SP1 remained unchanged in CMs throughout all time points (Supplementary Fig. 12b). Hence, in CMs, the Ago2 mRNA expression was coordinated with miR-320 expression throughout all time points. However, in CFs, Ago2 seemed to directly regulate the stability of miR-320 at the early time points after TAC via the post-transcriptional manner, while at the later time points, miR-320 was also transcriptionally regulated, which might be partly explained by the decreased SP1. The “temporal and spatial variation” nature of TFs, Ago2 and miRNAs during HF are intriguing subjects for future studies.

Recently, a few studies indicated that CMs and CFs, even macrophages and ECs were able to interact with each other in the pathophysiology of cardiac hypertrophy. The activation of vascular endothelial growth factor receptor 2 (VEGFR2)-Notch in ECs induced CMs hypertrophy via paracrine signaling.^36^ Moreover, the mutation of the RAF1 gene in CMs could activate CFs and then augment fibrosis.^37^ Cardiac macrophages were capable of secreting IL-10 and motivating CFs, which in turn boosted collagen deposition, and induced impaired heart function.^38^ CF-derived miR-21-3p mediated CMs hypertrophy by targeting the proteome profiling identified sorbin and SH3 domain-containing protein 2 (SORBS2) and PDZ and LIM domain 5 (PDLIM5).^39^ Interestingly, our data illustrated the complex crosstalk between CFs and CMs that miR-320 treated CFs were able to indirectly affect CMs function, but not vice versa. Notably, miR-320 itself was unable to transfer from CFs into CMs, nor from CMs into CFs under Ang II treatment. Interestingly, a cluster of Ang II-induced dysregulated proteins in the supernatant were rescued by miR-320 transfection in CFs. These proteins secreted from miR-320 transfected CFs might regulate the expression of CMs hypertrophy markers under stress, which are intriguing subjects for further study.

Notably, the inhibition of miR-320 in CFs failed to exacerbate the heart dysfunction in TAC mice. We reasoned that miR-320 was significantly decreased during HF, therefore miR-320 inhibition was unable to antagonize an already impaired miR-320-mediated signals. In addition, lowering miR-320 may require an extended period to observe a measurable alteration in CFs.

IFITM1 overexpression decreased p21 transcription and contributed to breast cancer progression.^40^ Meanwhile, some studies indicated that IFITM1 played a key role in the radio-resistance of oral neoplasm through the pSTAT3/p-p21 pathway.^41^ PLEKHM3 could potentially function as a scaffold protein for PKB during differentiation through PI3K signaling cascade.^42^ In the current study, we found that miR-320 inhibited IFITM1 expression in CFs and target PLEKHM3 in CMs, which was involved in the process of cardiac remodeling. We noted that PLEKHM3 was exclusively downregulated by CM-specific miR-320 overexpression, while IFITM1 was specifically suppressed by CF-specific miR-320 overexpression. These data suggested that PLEKHM3 and IFITM1 might function in CMs and CFs, respectively. Moreover, “targeting gene rescue” assays showed that miR-320 mediated effects were abolished by PLEKHM3 and IFITM1 re-expression in CMs and CFs, respectively, indicating that miR-320 mediated effects were PLEKHM3- and IFITM1-dependent.

In fact, until now cardio-fibroblast-specific promoter has not yet been identified, the locations of generally recommended markers of CFs, including FSP-1, Periostin and Col1a1, are not unique to fibroblasts. For example, Periostin and Col1a1 were also expressed in CMs,^26,43^ despite a relatively less abundance compared to CFs. Herein lies the difficulty, as researchers had not reached a consensus definition of the fibroblasts and their markers.^44^ In the current study, FSP-1 was chosen mainly because CMs did not express FSP-1.^45^ However, FSP-1 are also expressed in many other cardiac cell types, such as ECs and immune cells,^45^ therefore raises other concerns about whether the beneficial phenotype was uniquely from CFs rather than a mixed contribution from multiple cell types. Therefore, we took advantage of rAAV9, a virus vector reportedly targeting CMs and CFs,^28,46^ while the transfection efficiencies of rAAV9 on cardiac ECs, vascular smooth muscle cells and immune cells were not revealed. Interestingly, we noted that GFP signal was clearly detected in CFs while undetectable in other cell types treated with rAAV9-FSP1-GFP. In contrast, pAAV9-FSP1-GFP plasmid transfection was able to induce GFP expression in vascular smooth muscle cells (Supplementary Fig. 13a–c). In vivo co-staining of cell type-specific markers in the heart further supported the transfection of rAAV-FSP1-GFP in CFs but not in CMs, ECs, or immune cells (Supplementary Fig. 6a, b). On the other hand, FSP1-promoter might target fibroblasts from other tissues. To avoid this, we took advantage of the proper dosage of rAAV9 system, which showed cardiac specificity with 1 × 10^11^ vector copy numbers, while a higher dosage of rAAV9 vector copy numbers was able to translocate into the liver, the muscle, the pancreas, etc.^47^ In the current study, mice were treated with rAAV9-FSP1-system at 1 × 10^11^ vector copy numbers to achieve the heart specific targeting. In vivo immunofluorescence revealed that GFP signal was specifically detected in the heart but not in other organs of mice treated with rAAV9-FSP1-GFP (Supplementary Fig. 14a). These data suggested that FSP-1 promoter alone might be insufficient to specifically target CFs, however, rAAV9-system carrying FSP-1 promoter significantly improved CFs targeting specificity. In contrast, transgenic mice under the control of FSP-1 promoter might affect the fibroblasts in various organs of the whole body. Moreover, we evaluated the location of EdU as well as cell type-specific markers, and found EdU signals in CF-marker positive cells but not in CM-marker positive cells, indicating that miR-320 induced CFs proliferation was not a contamination from CMs (Supplementary Figs. 3g and 15a).

In summary, we demonstrated that miR-320 played different roles in CMs and CFs in the pathological process of HF, respectively, which indicated that cell-type-specific investigations and related treatments should receive more attentions.

## Materials and methods

An expanded version of the Materials and methods section, including detailed experimental procedures on animals, a list of PCR primers and antibodies, are presented in the online-only Supplementary Data.

### Construction of recombinant adeno-associated virus

We used the recombinant adeno-associated virus (rAAV) system (type 9) to manipulate the expression of miR-320 in vivo. The rAAV system was a gift from Dr. Xiao Xiao (University of North Carolina at Chapel Hill). Moreover, we employed a cardiac troponin T (cTnT) promoter to express miR-320 in CMs, whereas replaced the initial cytomegalovirus (CMV) promoter with fibroblast-specific protein-1 (FSP1) promoter to exclusively express miR-320 in CFs. Based on the mature sequence of hsa-miR-320a provided by miRBase (Accession: MIMAT0000510), oligonucleotides were designed as miR-320 and miR-320-TUD. The sequence of miR-random was designed by RiboBio (Guangzhou, China). The rAAVs were packaged by triple plasmid co-transfection, as described previously.^48^ The sequences are listed as Supplementary Table 7.

### Human heart samples

Left ventricular tissues from 10 end-stage HF patients were collected during heart transplantation. Control heart specimens were obtained from donors of heart transplantation who died in accidents. All donor grafts were donated after brain death to the Red Cross Society and allocated by the China Organ Transplant Response System according to Chinese laws.^49^ The characteristics of the patients and controls are listed in Supplementary Table S1. The plasma samples were obtained from another cohort of 32 normal controls and 32 HF patients, and the baseline characteristics are listed in Table S2. All procedures involving human samples were approved by the ethics committee of Tongji Hospital and Tongji Medical College and complied with the principles outlined in the Declaration of Helsinki. Informed consent was provided by the subjects or their family members under certain situation in the study.

### Statistical analysis

Data are shown as mean ± SEM. The Student’s *t* test and ANOVA were used among different groups. All calculations were performed by Prism (version 6; GraphPad Software, La Jolla, CA) and values with *P* < 0.05 were considered significant.

## Supplementary information

Supplementary Materials

## Data Availability

The datasets generated during and/or analyzed during the current study are available from the corresponding author on reasonable request.
