# Supplementary material for: The double face of miR-320: cardiomyocytes-derived miR-320 deteriorated while fibroblasts-derived miR-320 protected against heart failure induced by transverse aortic constriction
Source: Signal Transduct Target Ther. 2021 Feb 18;6:69. doi: 10.1038/s41392-020-00445-8 (PMC7890065; doi:10.1038/s41392-020-00445-8)
Supplement: Supplementary file 1 — Supplementary Materials [file 41392_2020_445_MOESM1_ESM.docx]

Supplementary Materials for

The double face of miR-320: cardiomyocytes-derived miR-320 deteriorated while fibroblasts-derived miR-320 protected against heart failure induced by transverse aortic constriction

Xudong Zhang^1#^, MD; Shuai Yuan^1#^, MD; Huaping Li^1#^, MD, PhD; Jiabing Zhan^1^, MD; Feng Wang^1^, MD, PhD; Jiahui Fan^1^, MD, PhD; Xiang Nie^1^, MD; Yan Wang^1^, MD, PhD; Zheng Wen^1^, MD, PhD; Yanghui Chen^1^, MD; Chen Chen^1*^, MD, PhD; and Dao Wen Wang^1*^, MD, PhD

Correspondence to: chenchen@tjh.tjmu.edu.cn; dwwang@tjh.tjmu.edu.cn

**This PDF file includes:**

Materials and Methods

Supplementary Text

Figures. S1 to S15

Tables S1 to S8

Materials and Methods

**Reagents**

Dulbecco’s modified Eagle’s medium (DMEM) and fetal bovine serum (FBS) were purchased from GIBCO (Grand Island, NY). Lipofectamine 2000 (Lipo 2000) reagent was obtained from Invitrogen (Carlsbad, CA). MiRNA-320 mimics, miRNA-320 inhibitor, biotin labeled miR-320, Cy3-labeled miR-320 and their relative controls were purchased from RiboBio (Guangzhou, China). The primers of miR-320 and U6 were also provided by RiboBio (Guangzhou, China). Real-time PCR primers of mRNA were synthesized by Tianyihuiyuan (Wuhan, China). Antibodies against GFP (Cat No: AE012) and Col1a1 (Cat No: A1352) were purchased from Abclonal (Wuhan, China). Anti-Cardiac Troponin T (Cat No: ab33589) was purchased from Abcam (Cambridge, MA). Anti-GAPDH (Cat No: 60004-1-Ig), anti-IFITM1 (Cat No: 60074-1-Ig) were purchased from Proteintech (Chicago, IL). Anti-Ago2 (Cat No: H00027161-M01) was purchased from Novus Biologicals (Beijing, China). Anti-PLEKHM3 (Cat No: 5487) was purchased from ProSci Incorporated (Poway, CA). Anti-ELF1 (Cat No: A03187-1) and anti-STAT1 (Cat No: A00036-2) were purchased from BOSTER (Wuhan, China). Ang II ELISA kit (Cat No: JM-02490M1) was purchased from JING MEI (Jiangsu, China). Polyvinylidene difluoride (PVDF) membranes were purchased from Millipore (Darmstadt, Germany). FITC-phalloidin (Cat No: P5282) and other chemical reagents were purchased from Sigma-Aldrich Company (Shanghai, China).

**Animals**

The research was approved by the Institutional Animal Research Committee of Tongji Medical College. All animal experimental protocols conformed to the US National Institutes of Health guidelines for the Care and Use of Laboratory Animals. Male C57BL/6 mice aged 8 weeks were purchased from the Experimental Animal Center of Hubei (Wuhan, China). For the purpose of manipulating miR-320 expression in CMs, mice were randomly divided into various groups as follows: sham, TAC, TAC+rAAV9-TNT-GFP, TAC+rAAV9-TNT-miR-320 and TAC+rAAV9-TNT-miR-320-TUD (n = 20/group). Meanwhile, in order to manipulate miR-320 expression in CFs, another 100 mice were randomly divided into groups as follows: sham, TAC, TAC+rAAV9-FSP1-miR-con, TAC+rAAV9-FSP1-miR-320 and TAC+rAAV9-FSP1-miR-320-TUD (n = 20/group). Each mouse received a single intravenous injection of 1 × 10^11^ corresponding virion particles in 100 μL saline solution via tail vein. Two weeks after the rAAV9 injection, transverse aortic constriction (TAC) was applied to induce pressure overload-induced cardiac hypertrophy. Briefly, mice were anesthetized with sodium pentobarbital (50 mg/kg, Sigma-Aldrich, Cat No: P3761) by intraperitoneal injection, and the aortic arch was accessible by blunt dissecting the second intercostal space. A 7-0 polypropylene suture was banded against a 27G needle around the aortic arch. Then the needle was carefully removed, muscle and skin were sutured layer by layer to close the chest cavity by 4-0 polypropylene suture. Sham mice underwent the similar surgical operation without aortic constriction. Eight weeks after the operation, all animals were anesthetized via intraperitoneal injections of a xylazine (5 mg/kg) and ketamine (80 mg/kg) mixture, and sacriﬁced subsequently. Tissue samples were obtained and frozen in liquid nitrogen, followed by storage at -80 °C.

**Echocardiography and hemodynamics**

Echocardiography analysis was measured by a 30-MHz high-frequency scanhead (VisualSonics Vevo770, VisualSonics, Toronto, Canada) as described previously.^1^ Hemodynamic measurements were performed by using a Millar Catheter System (Millar 1.4F, SPR835, Millar Instruments Inc, Houston, TX) as described previously.^2^

**Cell culture and transfection**

Rat myoblast H9c2 cells, mouse fibroblast NIH3T3 cells and the human cardiomyocyte line AC16 were obtained from American Type Culture Collection (ATCC) (Manassas, VA) and cultured in DMEM supplemented with 10% FBS. Mouse atrial cardiomyocyte tumor lineage HL-1 cells was also from ATCC (Manassas, VA) and were cultured in Claycomb Medium (Sigma, Shanghai, China) with 4 mM L-gutamine (Sigma, Shanghai, China), 100 μM norepinephrine (Sigma, Shanghai, China) and 10% FBS. Cells were cultured at 37 °C with a 95% air, 5% CO_2_ atmosphere.

Cells were transfected with miR-320 mimics (100 nM), miR-320 inhibitor (100 nM), or their negative control (100 nM), respectively, according to the manufacturer’s protocol of Lipofectamine 2000 (Invitrogen, Carlsbad, CA).

*In vitro* studies (expect for RIP-Seq) were performed in NRCMs or NRCFs, which had normal cell morphology and maintained many of the important markers and functions as *in vivo*. Specifically, several critical studies (such as miR-320 targets identification, CM-CF crosstalk transwell study, etc.) were repeated in multiple cell lines to explore the potential functional discrepancies among species.

**Isolation of cardiomyocytes and cardiac fibroblasts in adult mice**

Adult cardiac myocytes (CMs) and fibroblasts (CFs) were isolated from C57BL/6 mouse hearts as described previously.^3^ Briefly, the mouse was anesthetized with a mixture of 1% pentobarbital and heparin (100 U/mL) by intraperitoneal injection. The heart was quickly removed and perfused using a Langendorff system in Ca^2+^-free perfusion buffer at 37 °C containing NaCl 125 mmol/L, HEPES 15 mmol/L, KCl 5 mmol/L, taurine 6 mmol/L, MgCl_2_ mmol/L, glucose 10 mmol/L, 2,3-butanedion monoxine 7.5 mmol/L and the perfusion buffer was adjusted at pH 7.35-7.38 (at 25 °C). Subsequently, the perfusion buffer was substituted by enzymatic buffer (Ca^2+^-free perfusion buffer supplemented with 600 units/mL collagenase II). The perfusion pressure was constant at 120 cm H_2_O. Then the perfusion was accomplished while the drop rate sharply accelerated. Afterwards, the ventricle part was gently cut into small pieces with fine forceps in the same enzyme solution. The digestion was neutralized with DMEM containing 20% FBS. CMs were precipitated by gravity (30 min) and the supernatant containing CFs was gathered. CMs were resuspended in perfusion buffer containing 10% FBS and then allowed to deposit for 20 min. The two supernatants were mixed and both including CFs were centrifuged at 600 g for 10 min. The sediment was resuspended with DMEM supplemented with 10% FBS and laid on 6-well or 12-well plates.

**Isolation of cardiomyocytes and cardiac fibroblasts in neonatal rats**

Neonatal rat cardiac myocytes (NRCMs) and cardiac fibroblasts (NRCFs) were separated from 1- to 3-day-old Sprague Dawley rats as described previously.^4^ In brief, the hearts were cut into small pieces in ice-cold Hanks’ Balanced Salt Solution and then digested with 0.1% collagenase II and 0.08% trypsin at 37 °C. Every 8 minutes the supernatant containing the digested cells was transferred to a new sterile tube containing DMEM supplemented with 20% FBS and 1% penicillin/streptomycin. The remaining tissues were digested with fresh enzymatic solution for 5 to 6 times. Then the whole collected cell suspensions were filtered (70 μm cell strainer filter, BD) and centrifuged at 1200 g for 10 min, the pellet was resuspended in DMEM medium containing 10% FBS and 1% penicillin/streptomycin. Subsequently, the solution was adhered to tissue culture dishes for 2 h at 37 °C in a 5% CO_2_ incubator to get the cardiac fibroblasts and the supernatant, supposed to be cardiomyocytes, was counted to do the further experiment. The cardiomyocytes were cultured in DMEM supplemented with 10% FBS, BrdU (0.1 mM, to inhibit fibroblasts proliferation), and 1% penicillin/streptomycin. The primary cells were confirmed by immunofluorescence staining with antibodies directed against cardiomyocyte-specific marker α2-actinin (ACTN2), fibroblast-specific marker P4HB, and endothelial cell marker platelet/endothelial cell adhesion molecule CD31 as described previously.^5^

**RNA isolation and quantitative real-time polymerase chain reaction (qRT-PCR)**

Total RNA was obtained by Trizol (Invitrogen, Carlsbad, CA) and reversely transcribed to cDNA using the first-strand cDNA synthesis kit (Thermo Scientific, Carlsbad, CA) according to the manufacturer’s instructions. The expression of miR-320 and mRNA was quantified by qRT-PCR using the SYBR® Select Master Mix (Life Technologies, Carlsbad, CA) based on a 7900HT FAST real-time PCR system (Life Technologies, Carlsbad, CA). U6 was used as endogenous control to miRNA, while GAPDH was applied to endogenous control to mRNA. Each sample has three replicate measurements. Relative expression levels were analyzed by the 2^-ΔΔct^ method as previously described.^6^ The primer sequences were showed in Supplementary Table 8.

**RIP sequencing by Ago2**

RIP assay was performed to evaluate Ago2 targeting at specific mRNA transcripts. Briefly, lysis of CMs and CFs transfected with miR-320 or mimic control were incubated with anti-Ago2 (Abnova, Cat No: H00027161-M01) control IgG at 4 °C for overnight in an inverse rotator. Following that, the Pierce™ protein A/G 1 Magnetic Beads (Thermo Fisher Scientific, Cat No:88802) was added to the reaction and gently vortexed. After standard wash, the immunoprecipitated RNA was eluted and purified with PCR Purification Kit (Qiagen). Input groups (mimic-random versus miR-320 mimic) and IP-Ago2 groups (mimic-random versus miR-320 mimic) were subjected to mRNA-sequencing. RNA sequencing was conducted by Personalbio (Shanghai, China). P < 0.05 and fold-change ≥ 2 between IP-Ago2 groups (mimic-random versus miR-320 mimic) were considered as statistically significant.

**Western blot**

Protein samples from cells or mice heart tissues with different treatments were collected and homogenized. Lysates (20 ug protein/lane) were separated by 10% (wt/vol) acrylamide gel SDS-PAGE electrophoresis and then were transferred to a polyvinylidene ﬂuoride (PVDF) membrane. Subsequently, 5% bovine serum albumin was used to block non-speciﬁc sites for 2 h at room temperature. Then the membranes were incubated with corresponding primary and secondary antibodies, the protein bands were visualized by enhanced chemiluminescence kit according to the manufacturer’s protocol. The intensities of individual bands were analyzed by Image J (National Institutes of Health Software, Bethesda, MD).

**Histological Analysis**

Mice heart tissues were ﬁxed with 4% paraformaldehyde, then embedded in parafﬁn, and cut into 4-mm-thick slices. And the slices were stained with hematoxylin–eosin (H&E), wheat germ agglutinin (WGA) or Sirius Red, respectively. The images were quantified by Image-Pro Plus Version 6.0 software (Media Cybernetics, Bethesda, MD).

**EdU assay**

The treated NRCFs were labeled with EdU (5-ethynyl-2'-deoxyuridine) according to the manufacturer’s protocol (Ribobio Corporation, Guangzhou, China).

**Fluorescein isothiocyanate (FITC)-Phalloidin Staining**

HL-1 cells were washed with PBS for 3 times and ﬁxed in 4% paraformaldehyde for 15 min, then followed by incubation with 0.1% Triton X-100 for 15 min. Afterwards, cells were incubated in FITC-phalloidin at 4 °C overnight away from the light, and finally photographed by a Nikon DXM1200 ﬂuorescence microscope and quantified by Image-Pro Plus Version 6.0 software (Media Cybernetics, Bethesda, MD).

**Co-immunoprecipitation with anti-Ago2 antibody**

Twenty-four hours after transfection with miR-320 mimics or miRNA random, cells were lysed and then immunoprecipitated with anti-Ago2 antibody or IgG (Santa Cruz Biotech) using protein G Sepharose beads (Santa Cruz Biotech), as described previously.^6^ The remaining products were extracted with TRIzol, and the levels of mRNA were quantified by real-time PCR.

**Dual luciferase assay**

For dual luciferase assay, 400 ng of pMIR-IFITM1 3’-UTR, pMIR-IFITM1 3’-UTR mutant, pMIR-PLEKHM3 3’-UTR, pMIR-PLEKHM3 3’-UTR mutant or the empty vector was transfected into HEK293 cells with 40 ng of pRL-TK plasmid (Promega, Madison, WI), respectively. Meanwhile, miR-320 mimics or miRNA random was co-transfected with those reporter plasmids at a final concentration of 100 nM. After 48 hours, luciferase activity was detected by Dual-Luciferase Reporter Assay System (Promega) according to the manufacturer’s protocol.

**Cell Viability**

NIH3T3 cells were cultured in 96-well plates, and 24 h later, miR-320 mimics or miRNA random was co-transfected with pcDNA3.1 empty vector or pcDNA3.1-IFITM1 vector. Twenty-four hours later, 10 mL CCK8 reagent (Beyotime, Shanghai, China) was added per well, and the cells were cultured at 37 °C for 0.5, 1, and 2 h, respectively. The absorbance was tested at 450 nm following the manufacturer’s recommendations. The images were analyzed by Image J (National Institutes of Health Software, Bethesda, MD).

**RNA fluorescence in situ hybridization (FISH)**

MiR-320 expression detection was performed using formalin-fixed and paraffin-embedded tissue specimens as previously described.

**Enzyme linked immunosorbent assay (ELISA)**

The concentration of Ang II in tissue lysis or plasma was measured according to the manufacturer’s protocol (JM-02490M1, JINGMEI, Jiangsu, China).

**Figure. S1.**

**Ang II protein levels in different tissues and the plasma of TAC mice.**

**a**

**
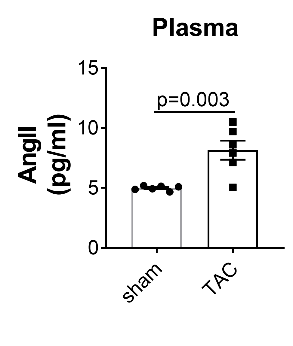

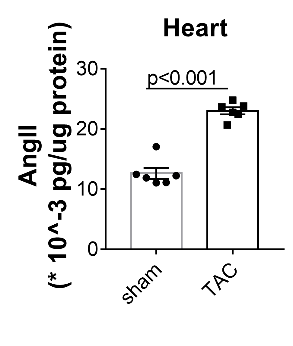

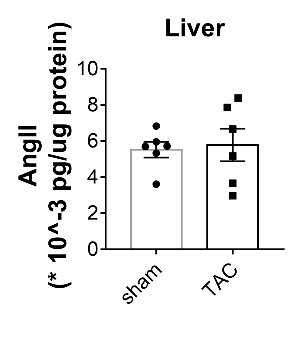

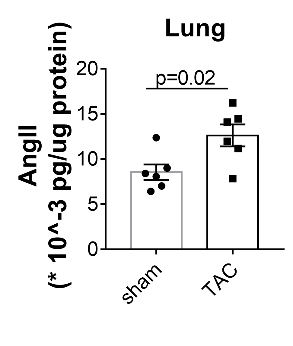
**

**
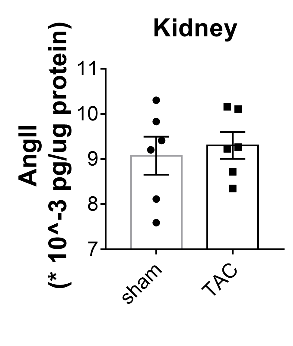
**

(a) Ang II protein levels in the plasma, the heart, the liver, the lung and the kidney were tested by ELISA. Sham (n = 6), TAC (n = 6). ELISA, enzyme linked immunosorbent assay.

**Figure. S2.**

**The purity of primary CMs and CFs in neonatal rats.**

**a**

**
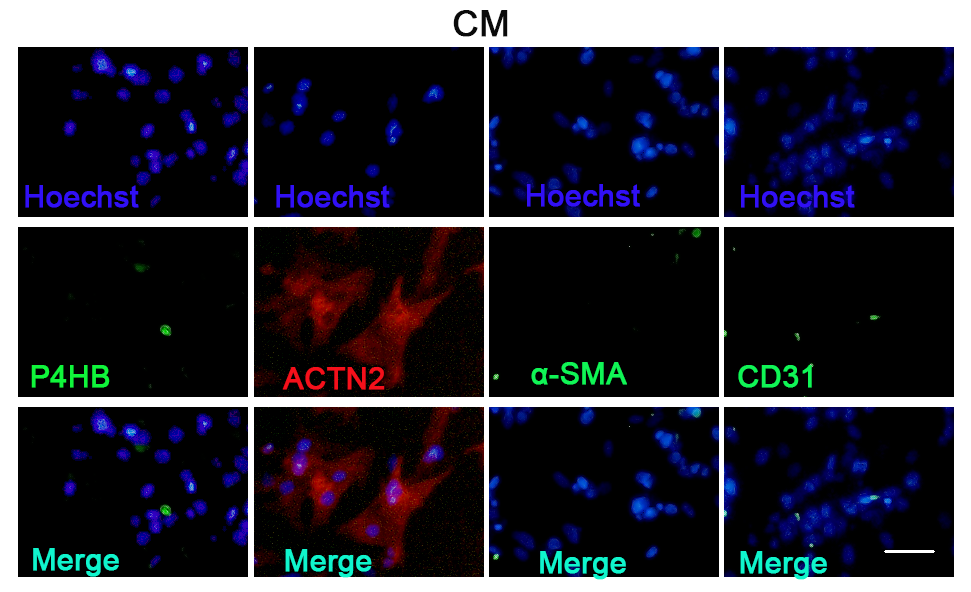
**

**b**

**
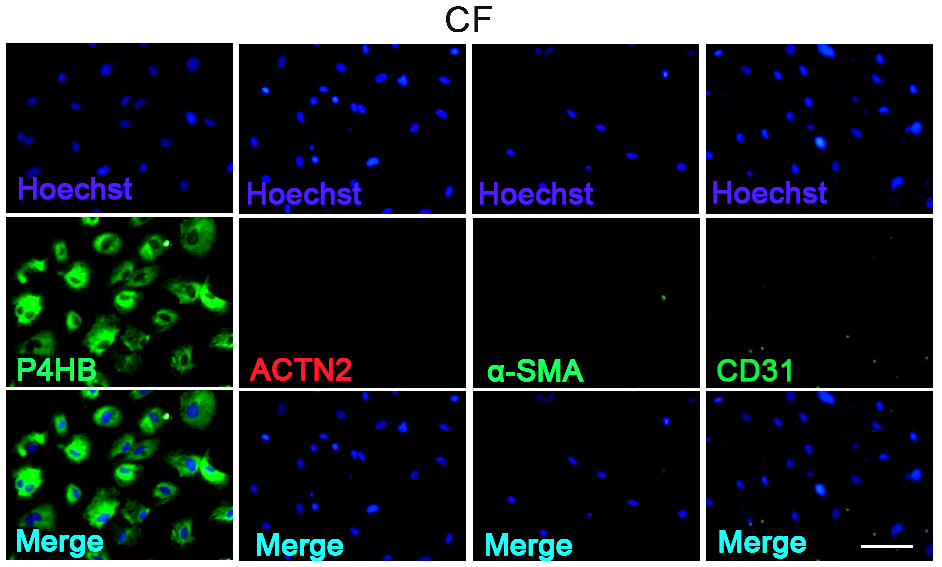
**

(a) CMs isolated from neonatal rats (NRCMs) were stained with Hoechst and antibodies against fibroblast-specific antigen prolyl-4-hydroxylase (P4HB), cardiomyocyte-specific marker α2-actin (ACTN2), smooth muscle-specific marker smooth muscle actin alpha 2 (α-SMA), and endothelial cell-specific marker platelet/endothelial cell adhesion molecule (CD31). Scale bar, 100μm. (b) CFs isolated from neonatal rats (NRCFs) were stained with Hoechst and antibodies against different cell type antigens. Scale bar, 50μm.

**Figure. S3.**

**Analyses of hypertrophy of NRCMs and fibrosis of NRCFs without Ang II treatment.**

**a b**

**



**

**c**

**
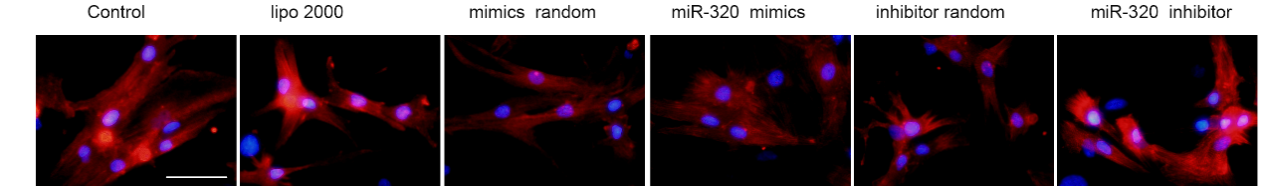
**

**d**

**
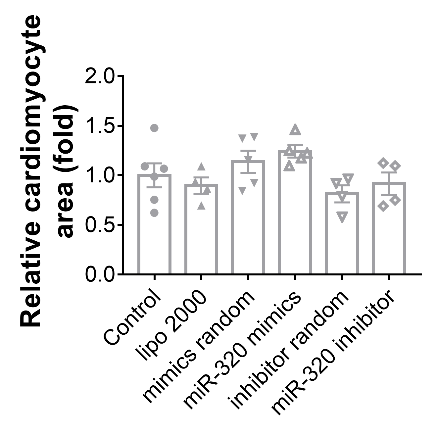
**

**e f**

**
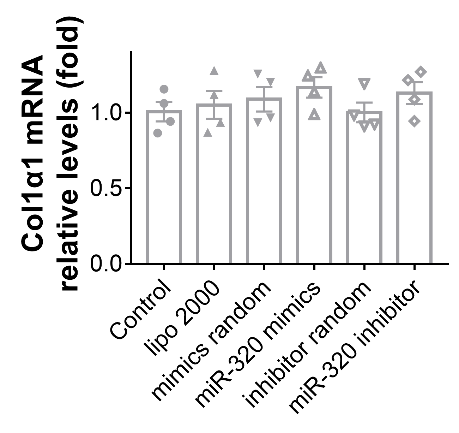

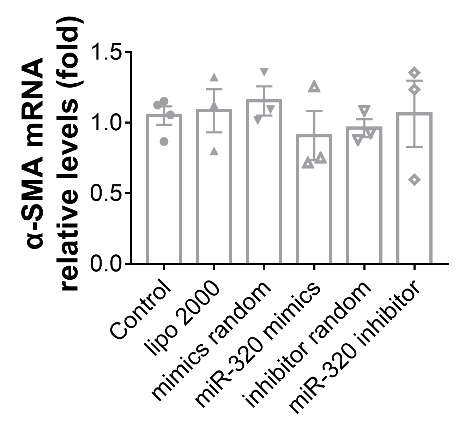
**

**g**

**
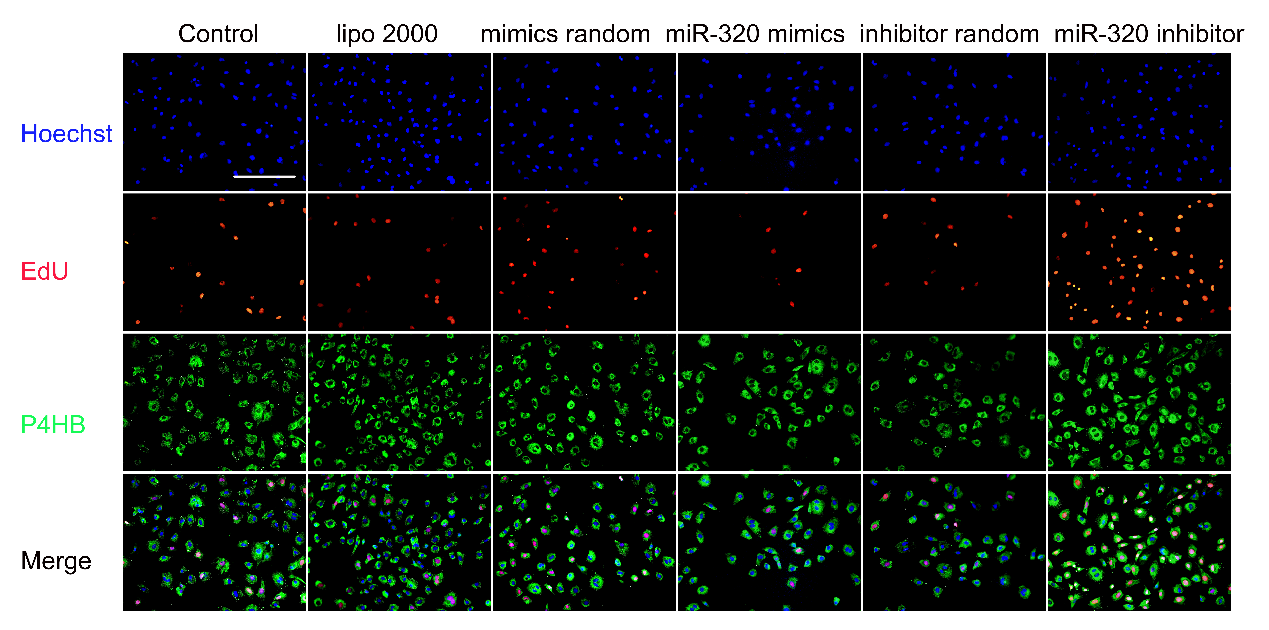
**

**h**

**
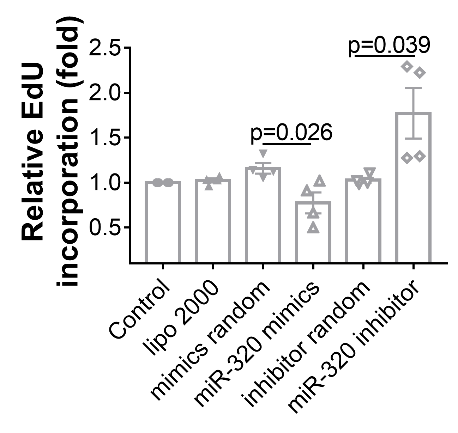
**

(a) ANP mRNA levels in CMs were quantified by qRT-PCR, relative to GAPDH (n = 3). (b) β-MHC mRNA levels in CMs were quantified by qRT-PCR, relative to GAPDH (n = 3). (c) Representative images of CMs areas stained by ACTN2 (red) and Hoechst (blue). Scale bar, 100 µm. (d) Quantitative analysis of cell sizes by Image-Pro Plus. (e) Col1α1 mRNA levels in CFs were quantified by qRT-PCR, relative to GAPDH (n ≥ 3). (f) α-SMA mRNA levels in CFs were quantified by qRT-PCR, relative to GAPDH (n ≥ 3). (g) Representative images of immunofluorescence staining for EdU (red), Hoechst (blue) and P4HB (green) in NRCFs with different treatments. Scale bar, 100 µm. (h) Quantitative analysis of EdU measured by Image J.

**Figure. S4.**

**Echocardiographic analysis of TAC mice at multiple time points.**

**a**

**
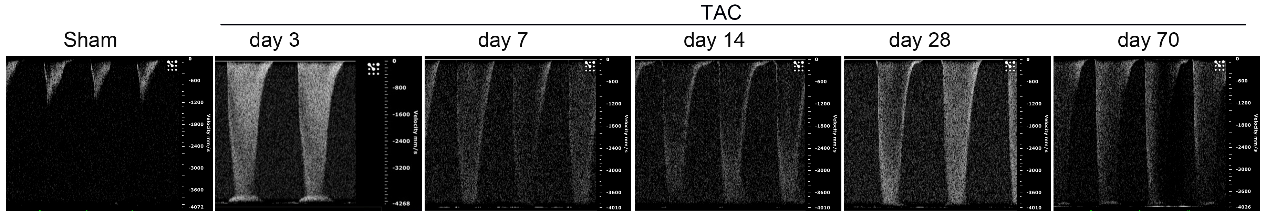
**

**b**

**
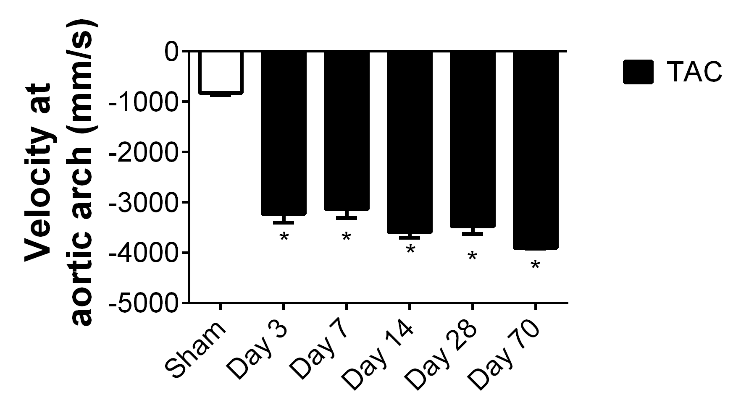
**

(a) Representative images of echocardiography of velocity at aortic arches in TAC mice. (b) Quantitative analysis of peak velocity in TAC mice aortic arches, *P < 0.05 vs. sham group.

**Figure. S5.**

**The purity of isolated CMs and CFs from adult mice.**

**
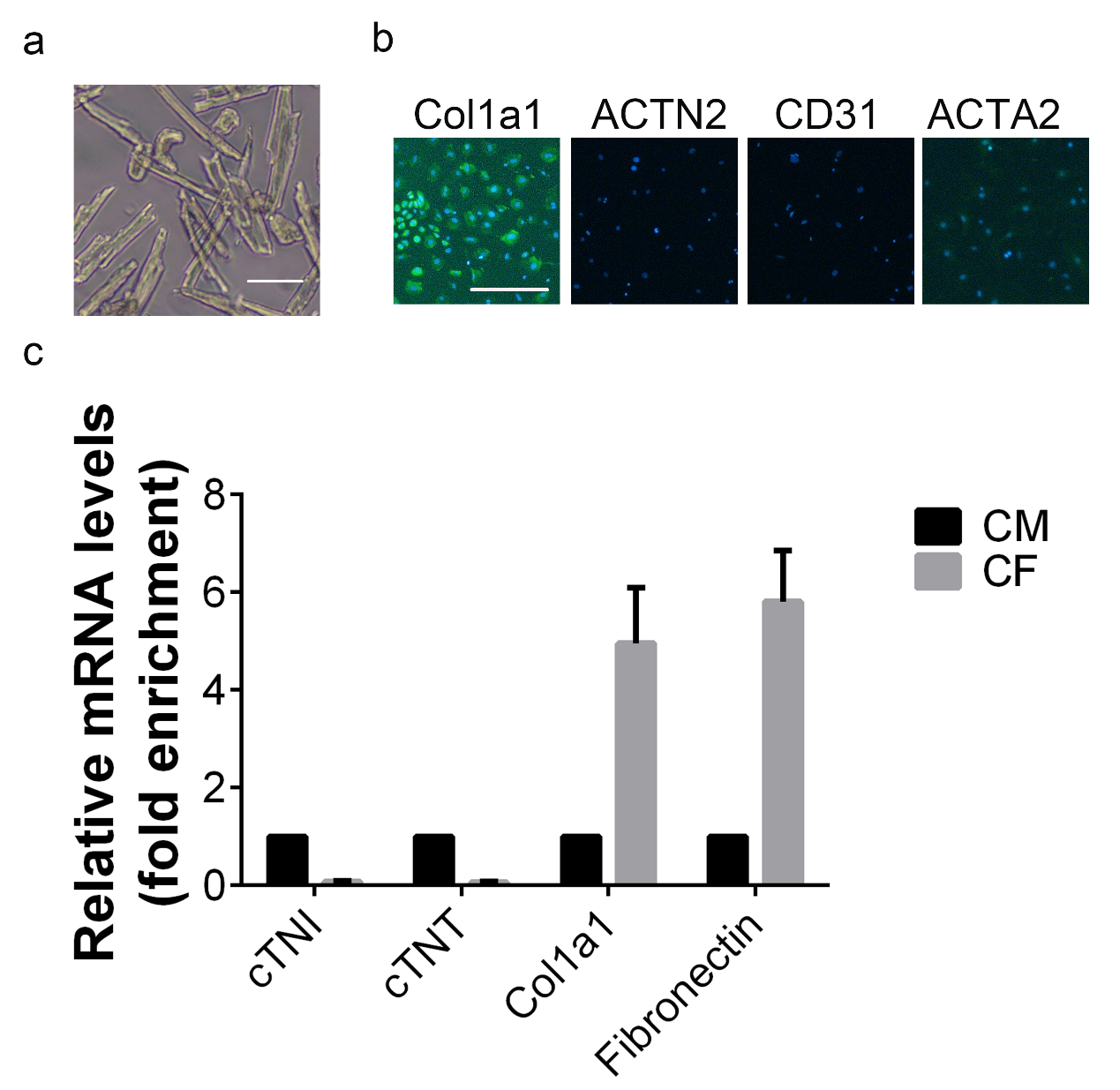
**

(a) The morphology of isolated CMs from adult mice. Scale bar, 100 µm. (b) CFs extracted from adult mice hearts were stained with DAPI and antibodies against fibroblast-specific antigen collagen type I alpha 1 chain (Col1a1), cardiomyocyte-specific marker α2-actin (ACTN2), endothelial cell-specific marker platelet/endothelial cell adhesion molecule (CD31) and smooth muscle-specific marker actin alpha 2 (ACTA2). Scale bar, 200μm. (c) The mRNA expression levels of cell type markers in isolated cells. CM, cardiac myocyte; CF, cardiac fibroblast; cTNI, cardiac troponin I; cTNT, cardiac troponin T.

**Figure. S6.**

**The specificity of rAAV vector with TNT promoter or FSP1 promoter in mice.**

**a**

**
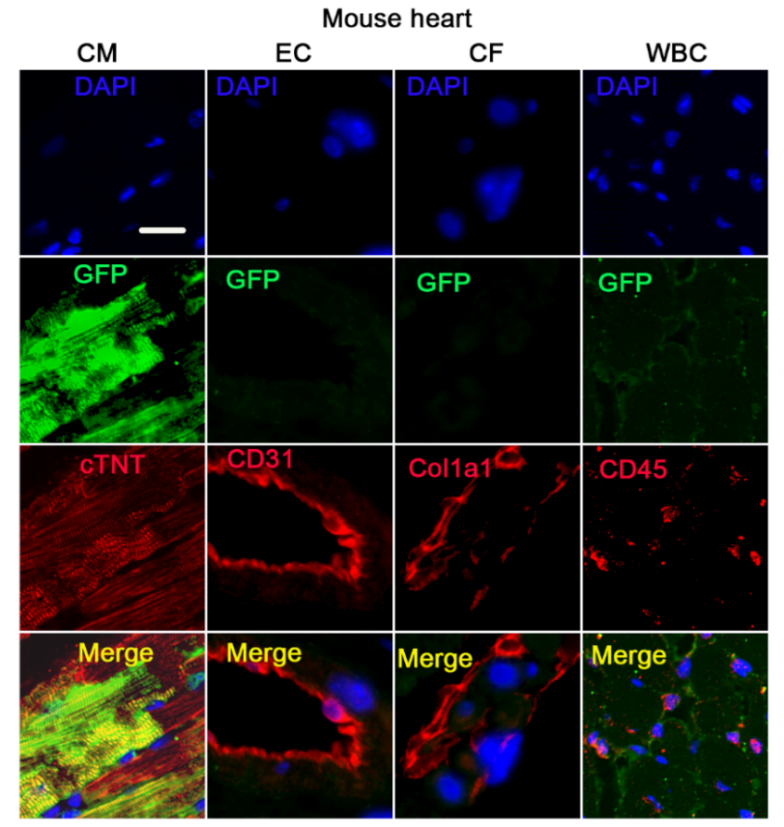
**

**b**

**
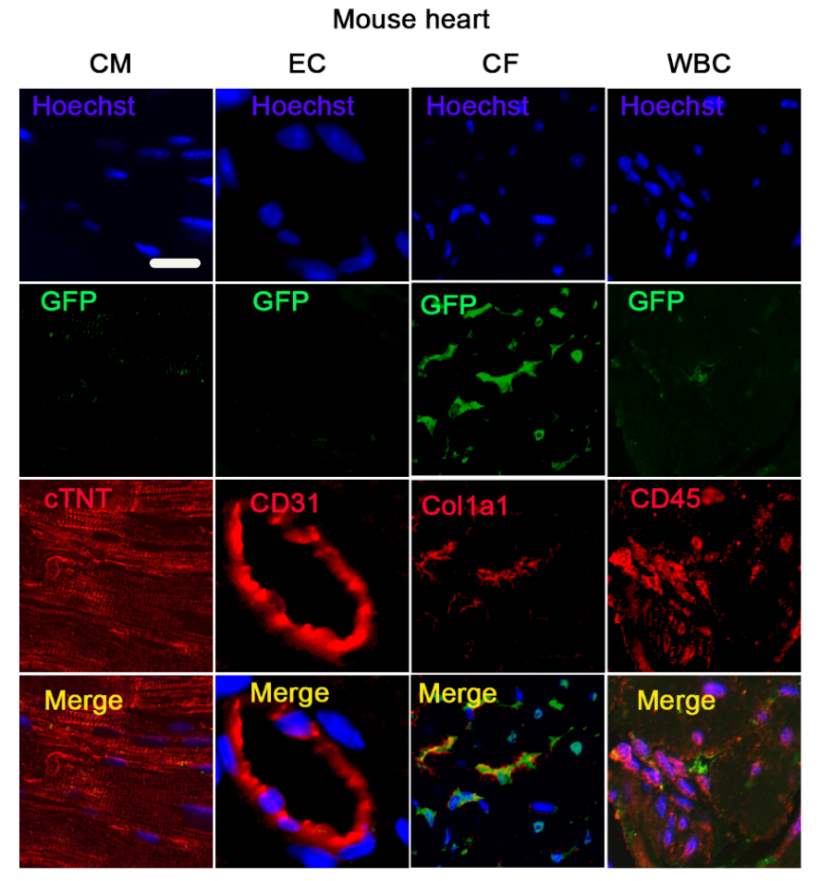
**

**c**

**
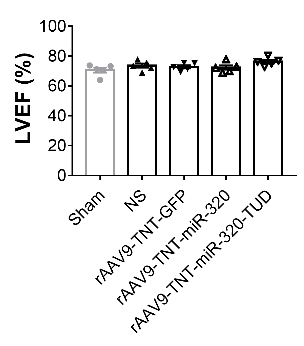

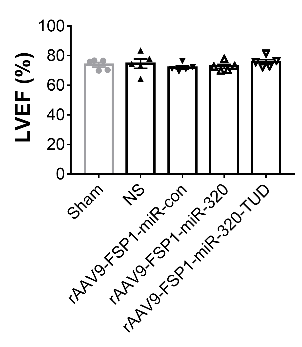
**

**d**

**
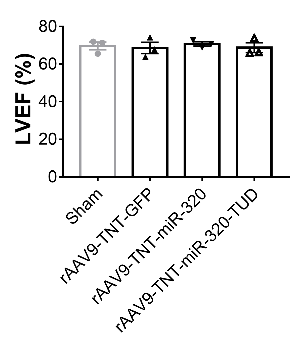

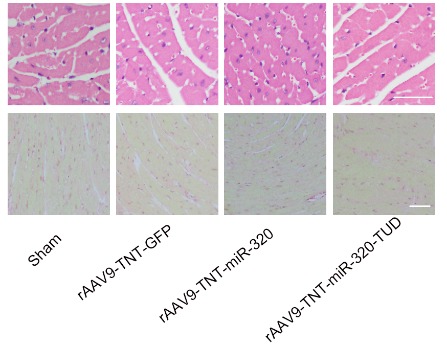
**

**e**

**
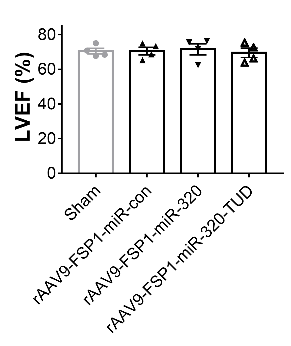

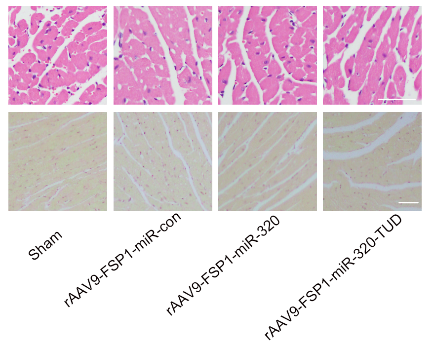
**

(a) In situ staining of GFP and cell type specific antigens (ACTN2, CD31, Col1α1, and CD45) in the heart tissues from rAAV9-TNT treated mice by immunofluorescence. Scare bar, 50 μm. (b) In situ detection of GFP and cell type specific antigens in the heart tissues from rAAV9-FSP1 treated mice by immunofluorescence. Scare bar, 50 μm. (c) LVEF% of rAAV-TNT (left) and rAAV-FSP1 (right) manipulated mice before TAC operation by echocardiography analysis, n = 6 per group. (d) LVEF% of rAAV-TNT manipulated mice after 10 weeks by echocardiography analysis (left). Representative images of H&E staining and Sirius Red staining of heart sections from mice (right), n = 3 per group. (e) LVEF% of rAAV-FSP1 manipulated mice after 10 weeks by echocardiography analysis (left). Representative images of H&E staining and Sirius Red staining of heart sections from mice (right), n = 4 per group. EC, endothelial cell; WBC, white blood cell.

**Figure. S7.**

**Co-culture assays of CMs and CFs.**

**a**

**
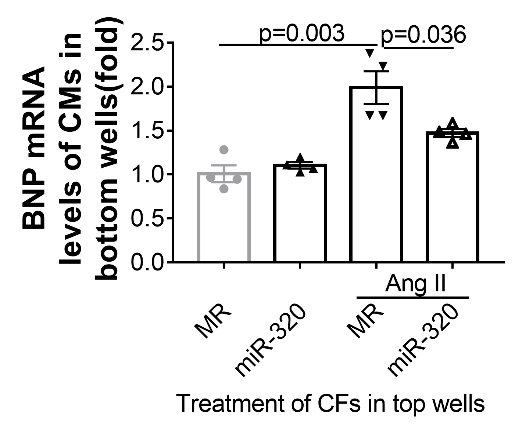

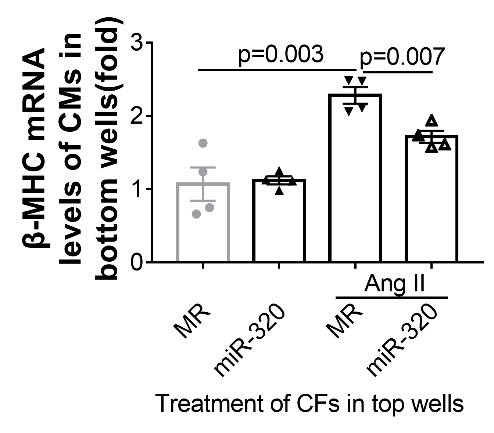
**

**b**

**
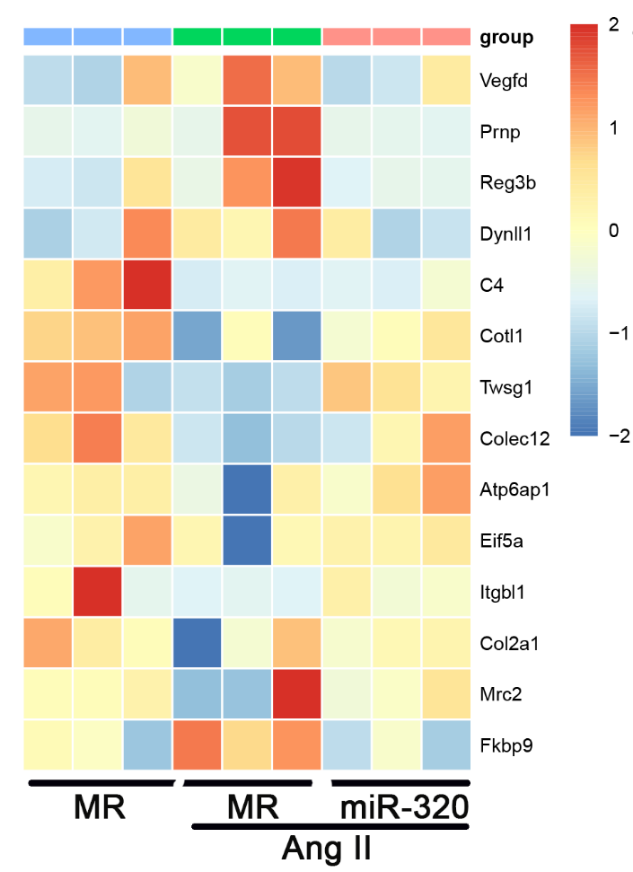
**

**c**

**
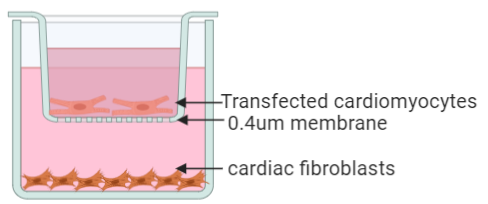
**

**d**


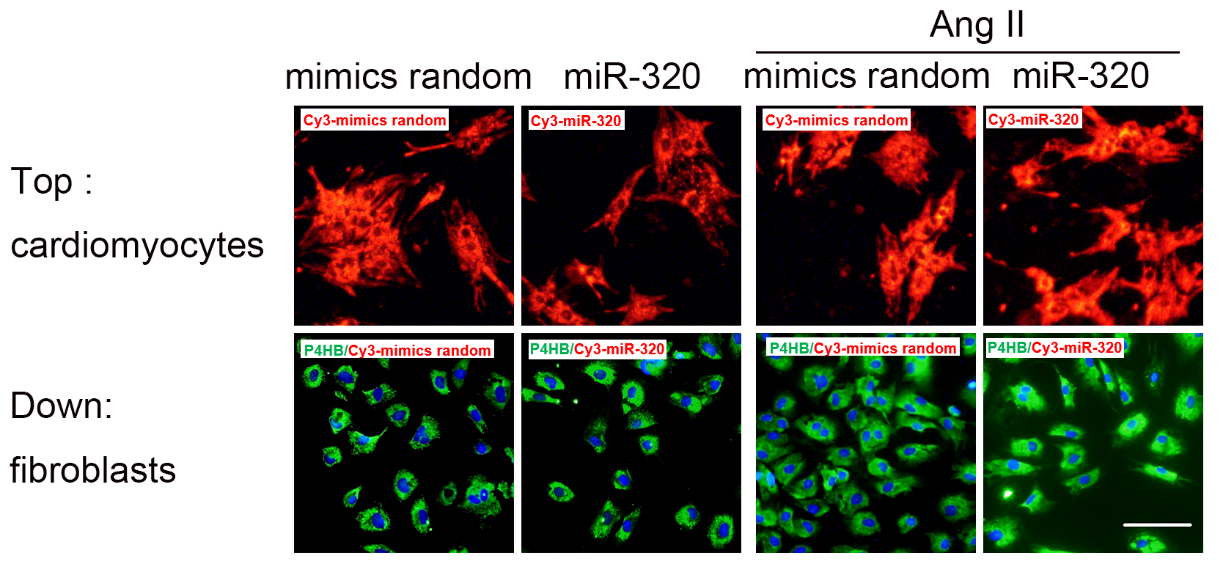


**e**

**
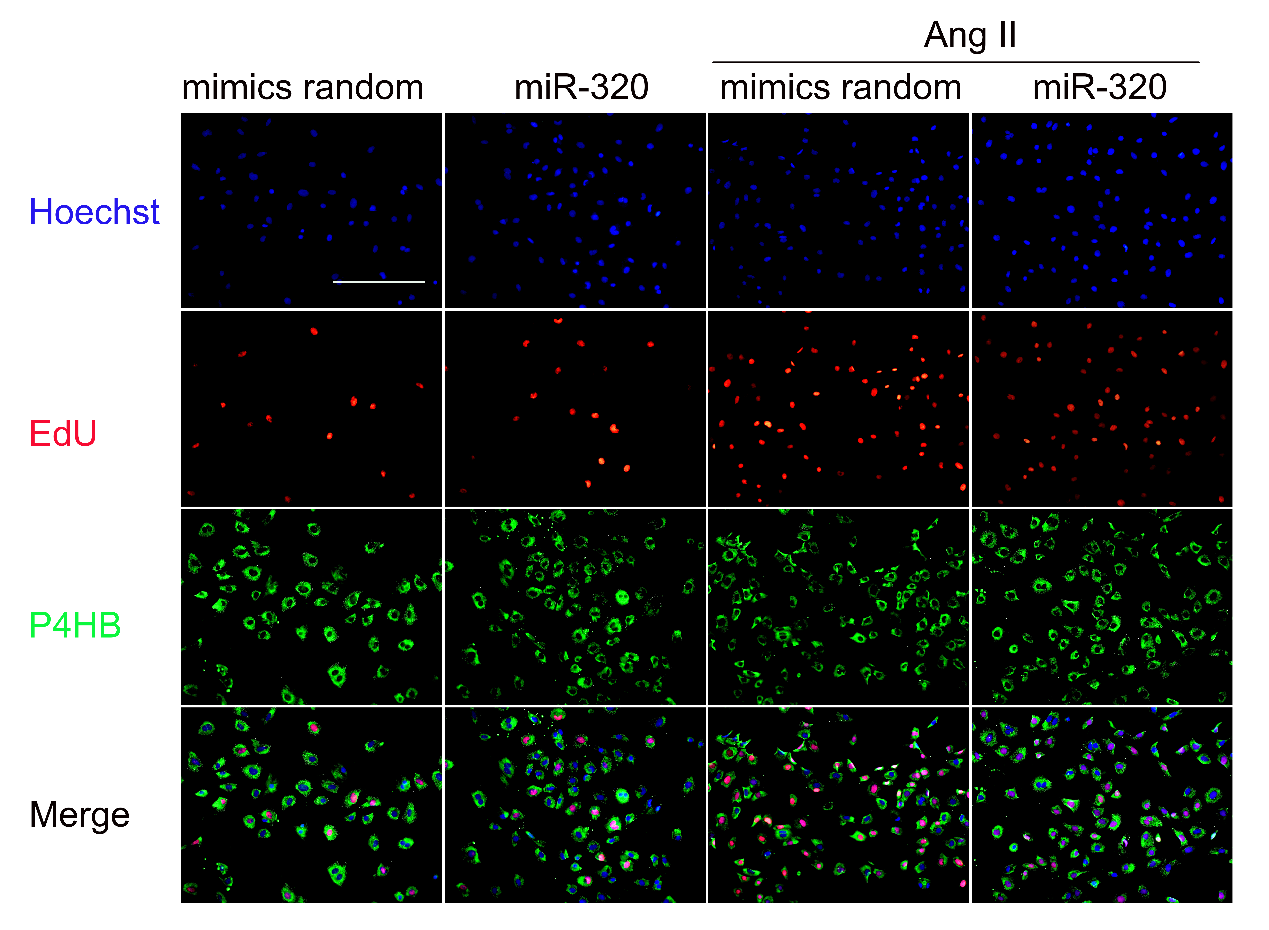
**

**
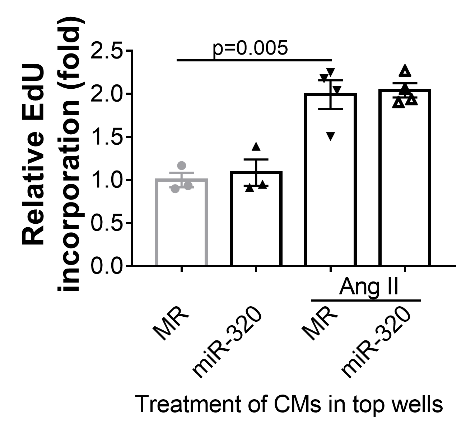
**

**f**

**
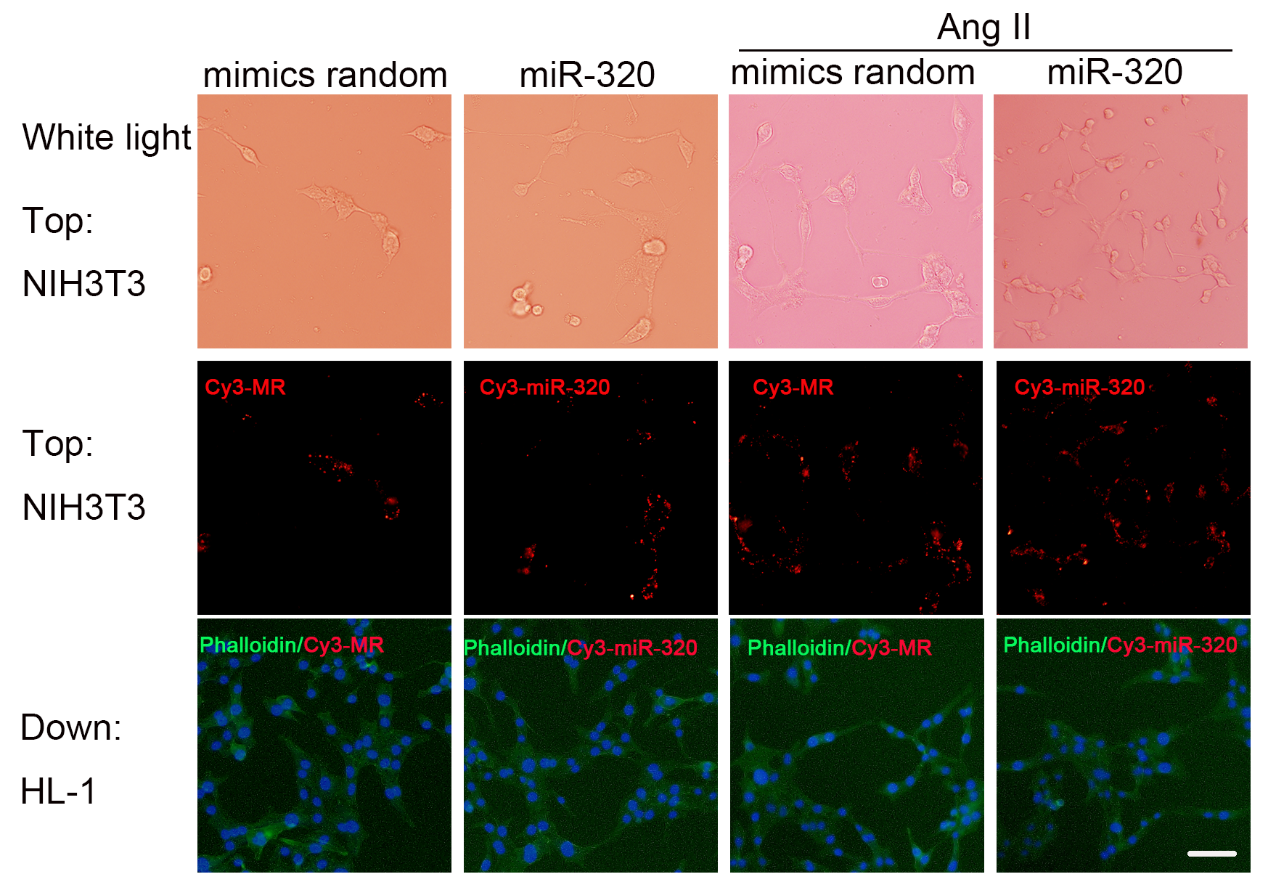
**

**g**

**
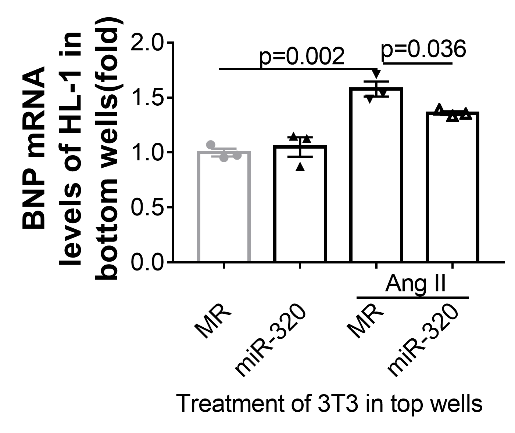

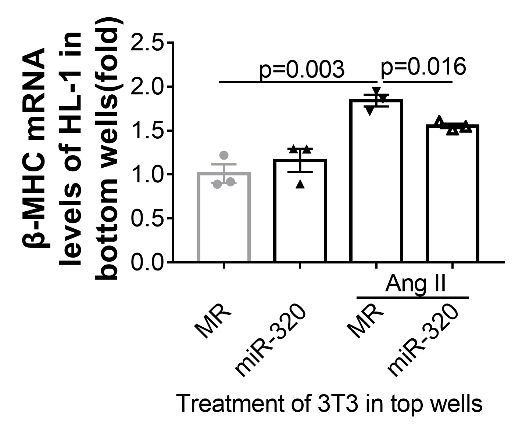
**

(a) BNP mRNA levels were quantified by qRT-PCR, relative to GAPDH (left, n = 4); β-MHC mRNA levels were quantified by qRT-PCR, relative to GAPDH (right, n = 4). (b) Heatmap of proteome profiling analysis of the supernatants were measured by LC-MS (n = 3 per group). (c) Schematic diagram of co-culture assay. Top well, NRCMs; lower chamber, NRCFs. (d) NRCMs in the top chamber were photographed by fluorescence microscope after being transfected with Cy3-labeled miR-320. P4HB staining of NRCFs in lower chamber. Scale bar, 100 µm. (e) Representative images of immunofluorescence staining for EdU (red), Hoechst (blue) and P4HB (green) in NRCFs with different treatments (up). Scale bar, 100 µm. Quantitative analysis of EdU gauged by Image J (down). (f) NIH3T3 cell lines in the top chamber were photographed by white light and fluorescence microscope after being transfected with Cy3-labeled miR-320. FITC phalloidin staining of HL-1 cell lines in lower chamber. Scale bar, 50 µm. (g) BNP mRNA levels were quantified by qRT-PCR, relative to GAPDH (left, n = 3); β-MHC mRNA levels were quantified by qRT-PCR, relative to GAPDH (right, n = 3).

**Figure. S8.**

**Reduced Ago2 did not affect pri-miR-320 expression in NRCMs and NRCFs.**

**a**

**
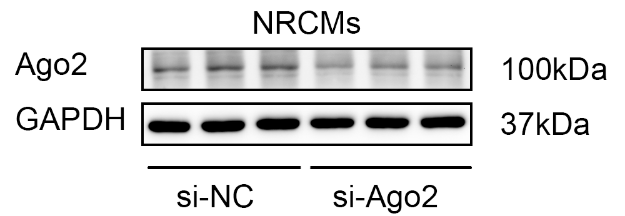


**

**b**

**
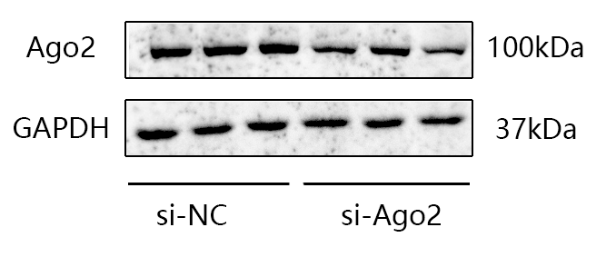


**

**c**

**
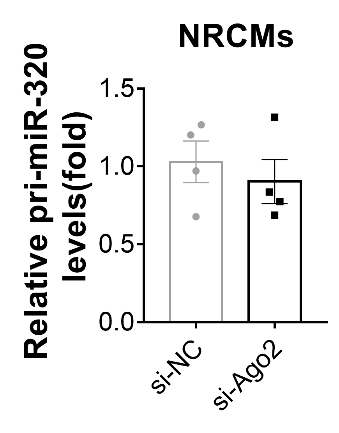

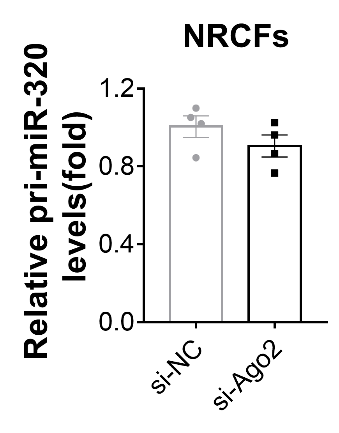
**

**d**

**
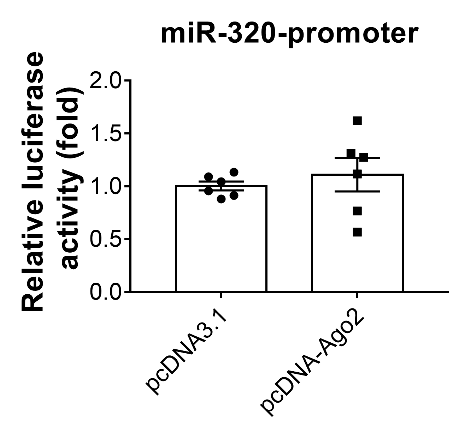
**

**e f**

**
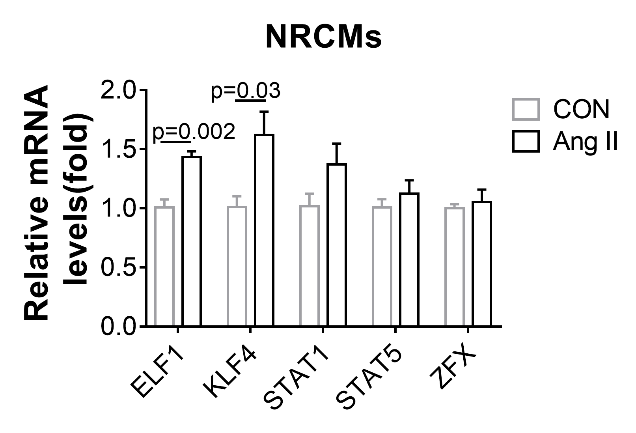

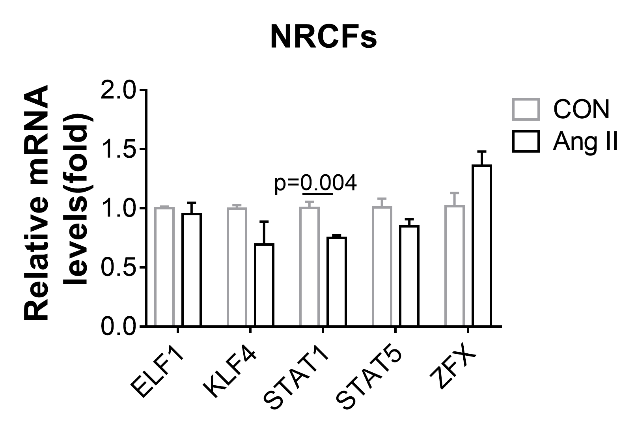
**

**g**

**
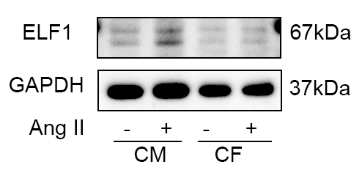

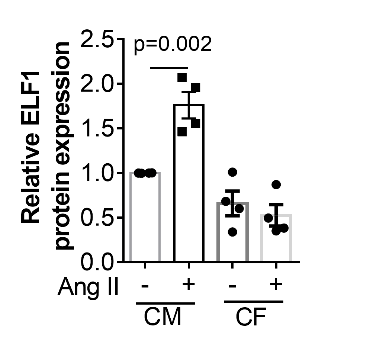
**

**h**

**
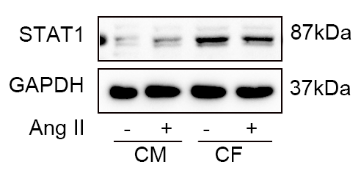

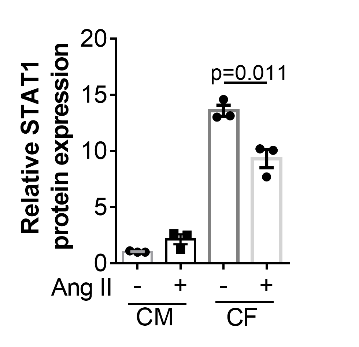
**

**i**

**
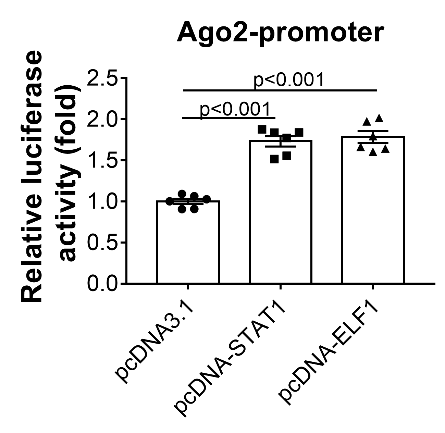
**

(a) Representative images showing protein expression levels of Ago2 and GAPDH in NRCMs after si-Ago2 transfection (left). Quantitative analysis Ago2 protein levels after normalization to GAPDH (right) (n ≥ 3). (b) Representative images showing protein expression levels of Ago2 and GAPDH in NRCFs after si-Ago2 transfection (left). Quantitative analysis Ago2 protein levels after normalization to GAPDH (right) (n ≥ 3). (c) Pri-miR-320 levels were quantified by qRT-PCR in NRCMs after si-Ago2 treatment, relative to GAPDH (left, n = 4); Pri-miR-320 levels were quantified by qRT-PCR in NRCFs after si-Ago2 treatment, relative to GAPDH (right, n = 4). (d) Regulation of Ago2 targeting miR-320 promoter was detected by luciferase reporter assays in HEK293T cells (n ≥ 3). (e) ELF1, KLF4, STAT1, STAT5 and ZFX mRNA levels in NRCMs 24 h after Ang II treatment (n = 4). (f) ELF1, KLF4, STAT1, STAT5 and ZFX mRNA levels of NRCFs 24 h after Ang II treatment (n = 4). (g) The protein levels of ELF1 in differently treated NRCMs and NRCFs were measured by Western blot analyses (left), and quantified by Image J (right) (n = 3). (h) The protein levels of STAT1 in differently treated NRCMs and NRCFs were measured by Western blot analyses (left), and quantified by Image J (right) (n = 3). (i) Regulation of ELF1 and STAT1 targeting Ago2 promoter was detected by luciferase reporter assays in HEK293T cells (n ≥ 3).

**Figure. S9.**

**MiR-320 affected PLEKHM3 in CMs and targeted IFITM1 in CFs.**

**a**

**
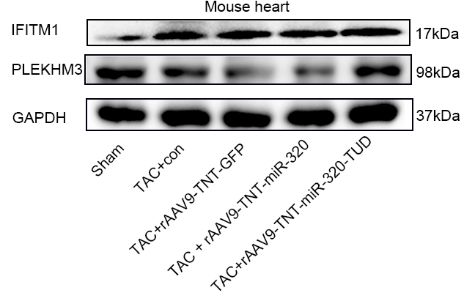

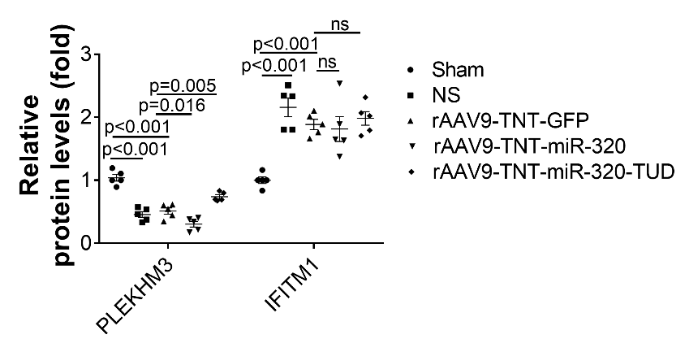
**

**b**

**
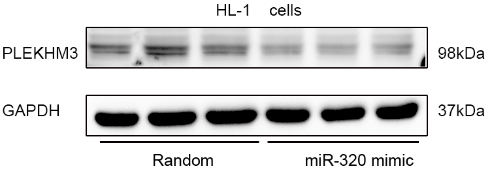

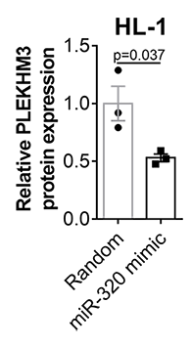
**

**c**

**
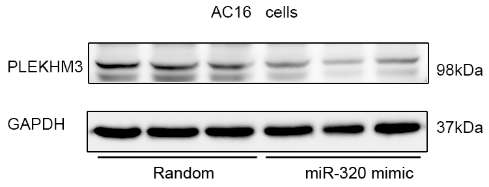

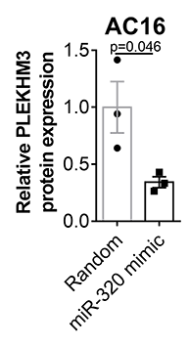
**

**d**

**
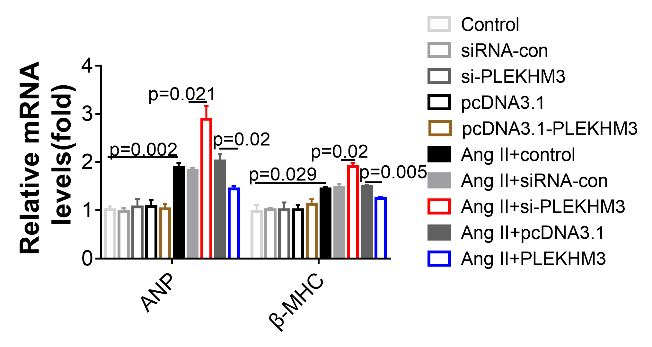
**

**e f**

**
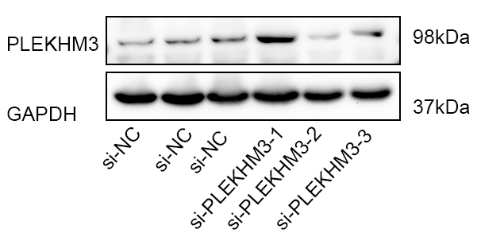

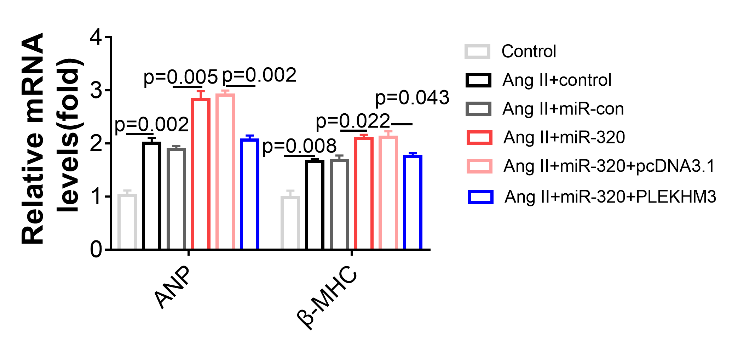
**

**g**

**
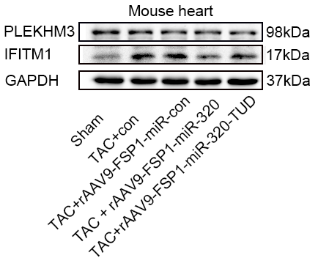

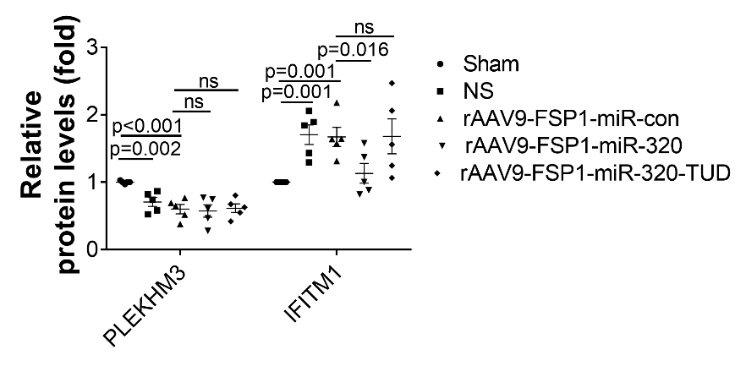
**

**h**

**
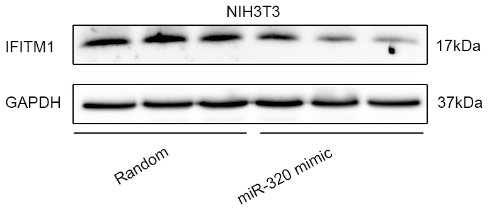

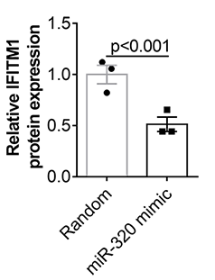
**

**i**

**
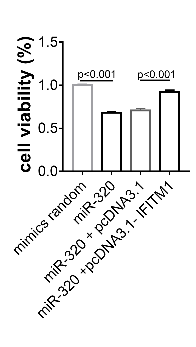
**

**j**

**
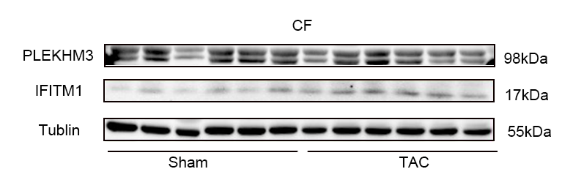

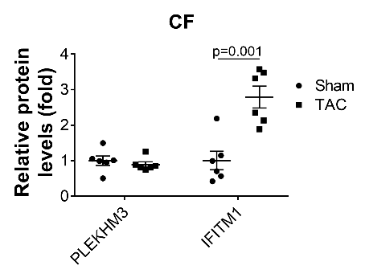
**

**k**

**
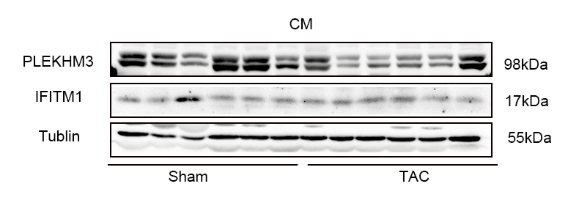

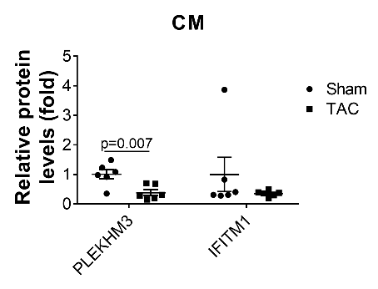
**

(a) The protein levels of PLEKHM3 and IFITM1 in the heart tissues of rAAV9-TNT treated mice were examined by Western blot analysis (up), and quantified by Image J (down). Sham (n = 5), TAC+con (n = 5), TAC+rAAV9-TNT-GFP (n = 5), TAC+rAAV9-TNT-miR-320 (n = 5), TAC+rAAV9-TNT-miR-320-TUD (n = 5). (b) The protein levels of PLEKHM3 in differently treated HL-1 cells were examined by Western blot analysis (left), and quantified by Image J (right) (n = 3). (c) The protein levels of PLEKHM3 in differently treated AC16 cells were examined by Western blot analysis (left), and quantified by Image J (right) (n = 3). (d) Expressions of cardiac hypertrophy markers in CMs with different treatments were measured by real-time PCR (n = 3). (e) The efficiency of siRNA on PLEKHM3 expression was detected by Western blot. (f) Expressions of cardiac hypertrophy markers in CMs with different treatments were measured by real-time PCR (n = 3). (g) The protein levels of IFITM1 and PLEKHM3 in the heart tissues of rAAV9-FSP1 treated mice were examined by Western blot analysis (up), and quantified by Image J (down). Sham (n = 5), TAC+con (n = 5), TAC+rAAV9-FSP1-miR-con (n = 5), TAC+rAAV9-FSP1-miR-320 (n = 5), TAC+rAAV9-FSP1-miR-320-TUD (n = 5). (h) The protein levels of IFITM1 in differently treated NIH3T3 cells were examined by Western blot analysis (left), and quantified by Image J (right) (n = 3). (i) Cell viability was tested by cell count-8 assays (n ≥ 3). (j) The protein levels of PLEKHM3 and IFITM1 in isolated CFs from adult mice heart tissues were examined by Western blot analysis (left), and quantified by Image J (right) (n = 6). (k) The protein levels of PLEKHM3 and IFITM1 in isolated CMs from adult mice heart tissues were examined by Western blot analysis (left), and quantified by Image J (right) (n = 6).

**Figure. S10.**

**MiR-29 expression could be also regulated by Ago2.**

**a b**

**
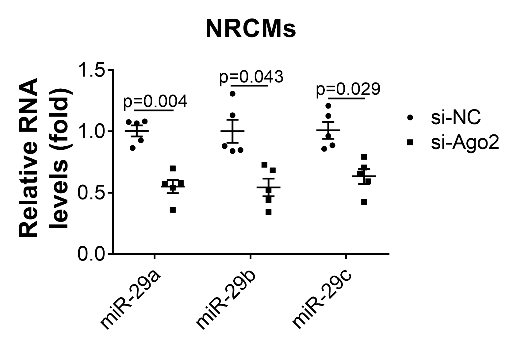

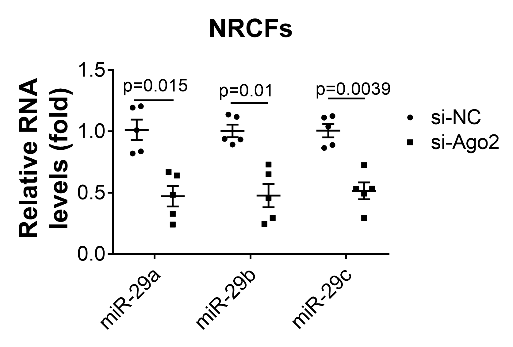
**

(a) Relative levels of miR-29a, miR-29b, and miR-29c in NRCMs transfected with si-Ago2 (n = 5). (b) Relative levels of miR-29a, miR-29b, and miR-29c in NRCFs transfected with si-Ago2 (n = 5).

**Figure. S11.**

**MiR-320 overexpression abolished Ago2 knockdown-mediated effects in CMs and CFs.**

**a**

**
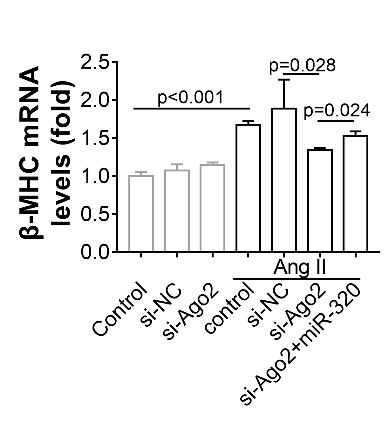
**

**b**

**
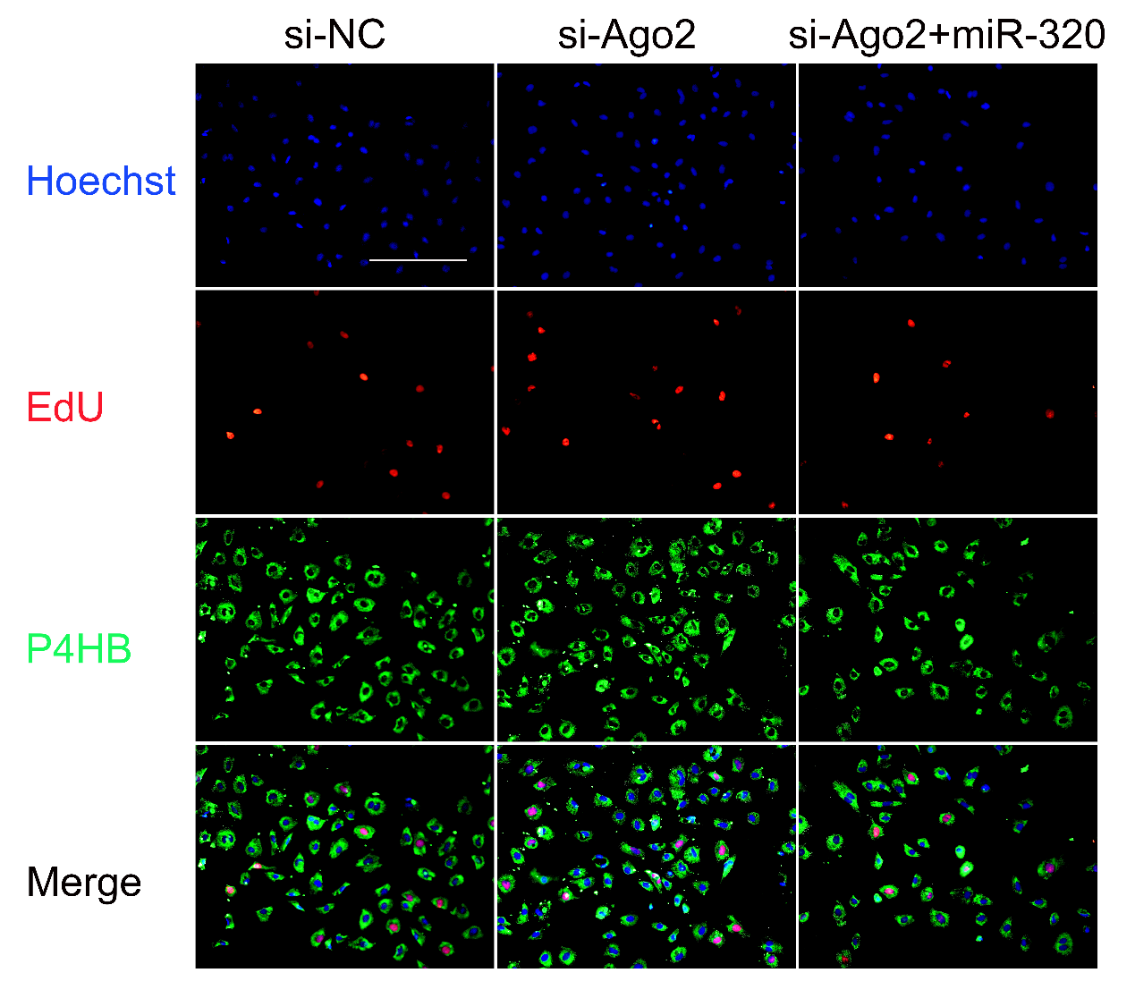
**

**
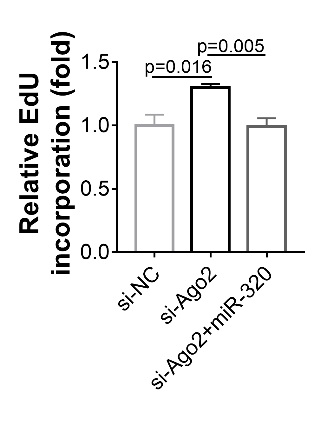
**

(a) Expressions of β-MHC mRNA in NRCMs with different treatments were measured by real-time PCR (n = 4). (b) Representative images of immunofluorescence staining for EdU (red), Hoechst (blue) and P4HB (green) in NRCFs with different treatments (up). Scale bar, 100 µm. Quantitative analysis of EdU was measured by Image J (down).

**Figure. S12.**

**SP1 mRNA levels and pri-miR-320 levels in TAC mice at different time points.**

**a**

**
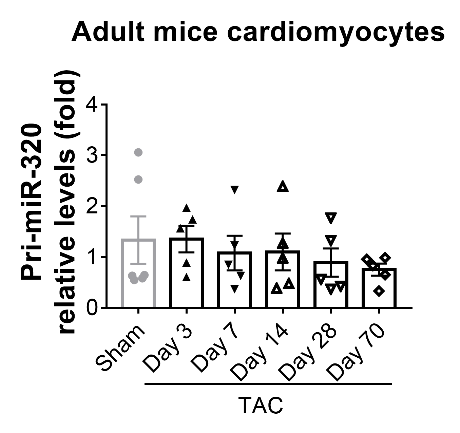

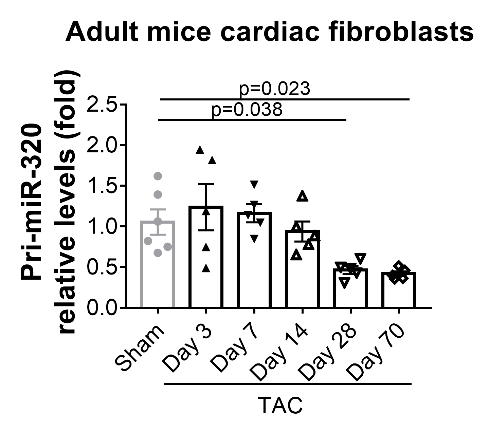
**

**b**

**
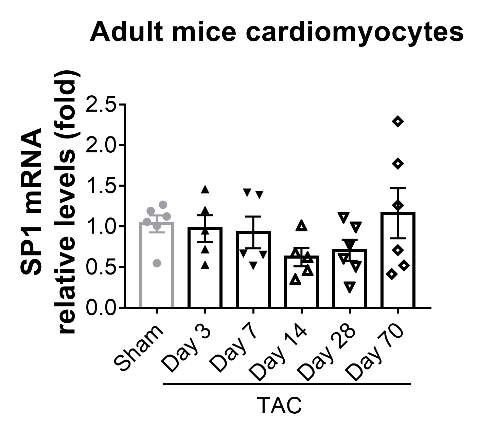

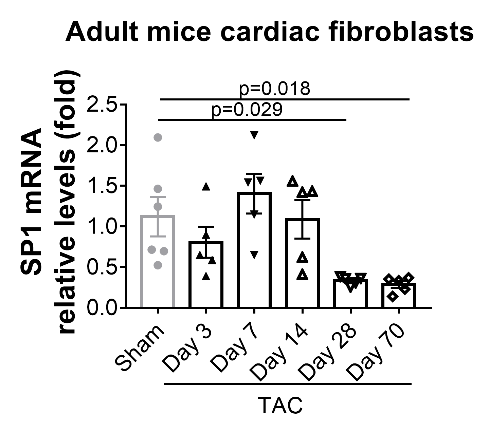
**

(a) The expressions of pri-miR-320 in isolated CMs (left) and CFs (right) were measured by real-time PCR. (b) The expressions of SP1 in isolated CMs (left) and CFs (right) were measured by real-time PCR. n ≥ 5 per group.

**Figure. S13.**

**The transfection efficiencies of pAAV9-FSP1-GFP plasmid, rAAV9-FSP1-GFP and rAAV9-TNT-GFP.**

**a**

**
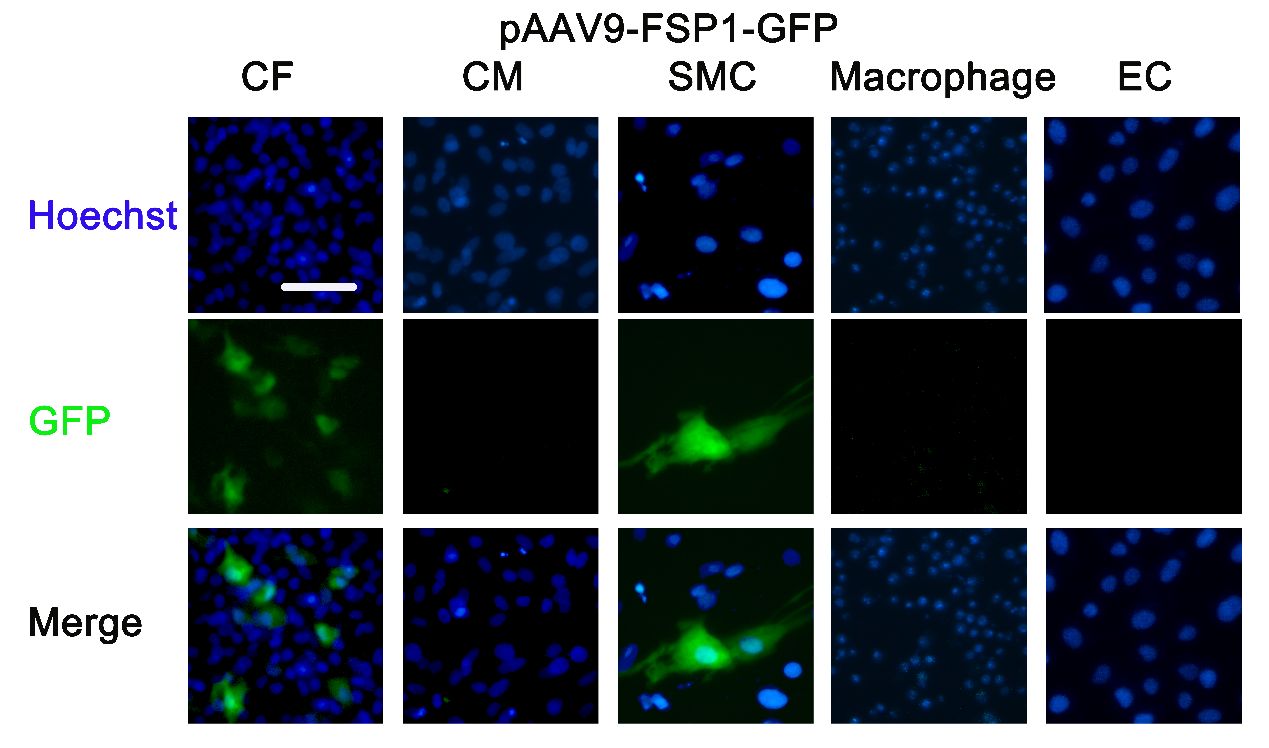
**

**b**

**
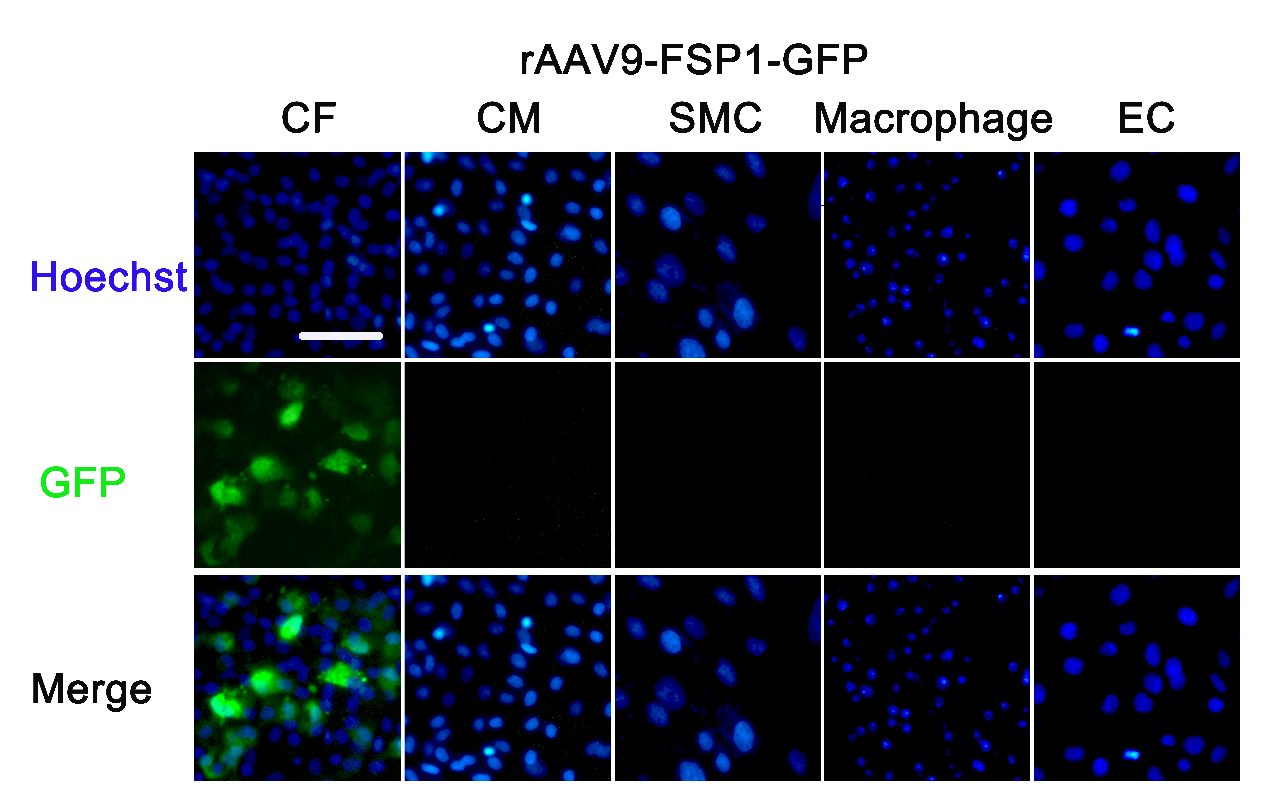
**

**c**

**
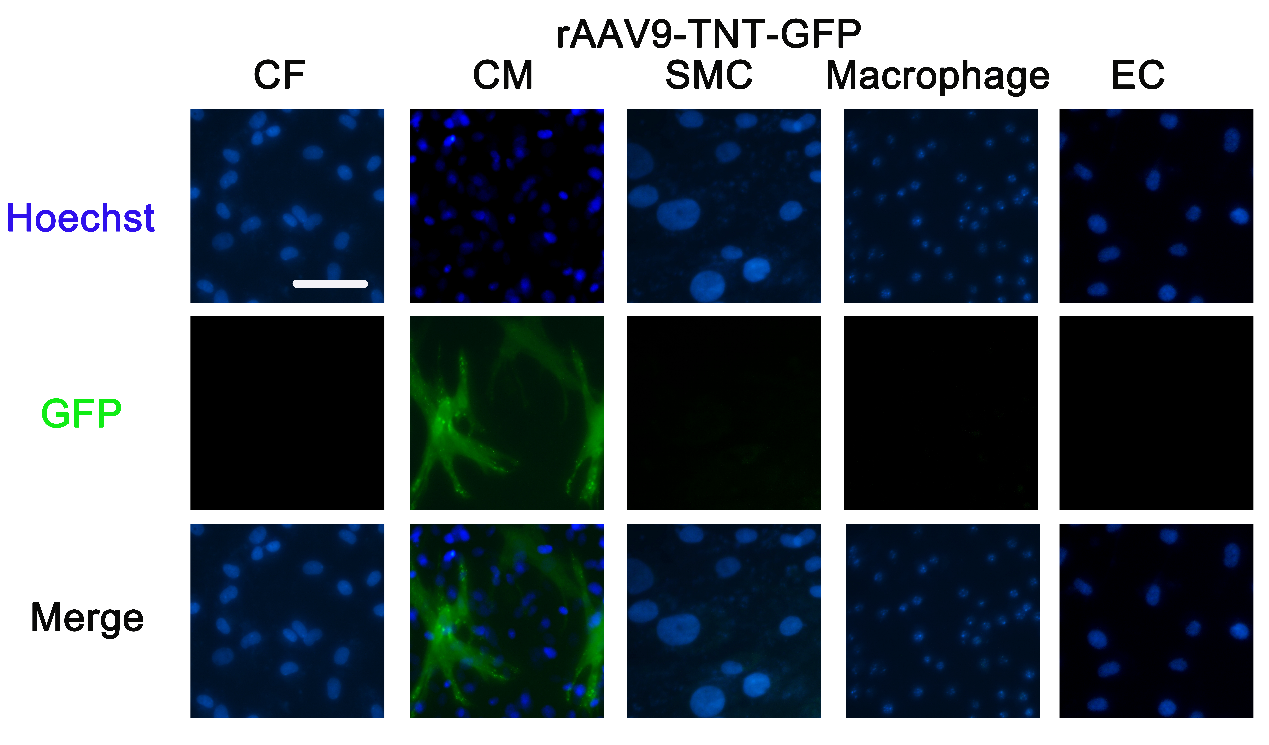
**

NRCFs, NRCMs, human smooth muscle cells (HSMC), RAW264.7 macrophages and human umbilical vein endothelial cells (HUVEC) were transfected with pAAV9-FSP1-GFP plasmid (a), rAAV9-FSP1-GFP (b) and rAAV9-TNT-GFP (c) for 7 days, cells were photographed by fluorescence microscope. Hoechst (blue), GFP (green). Scale bar, 50 µm.

**Figure. S14.**

**Immunofluorescence staining in the kidney, the liver, the muscle, the brain, and the heart.**

**a**

**
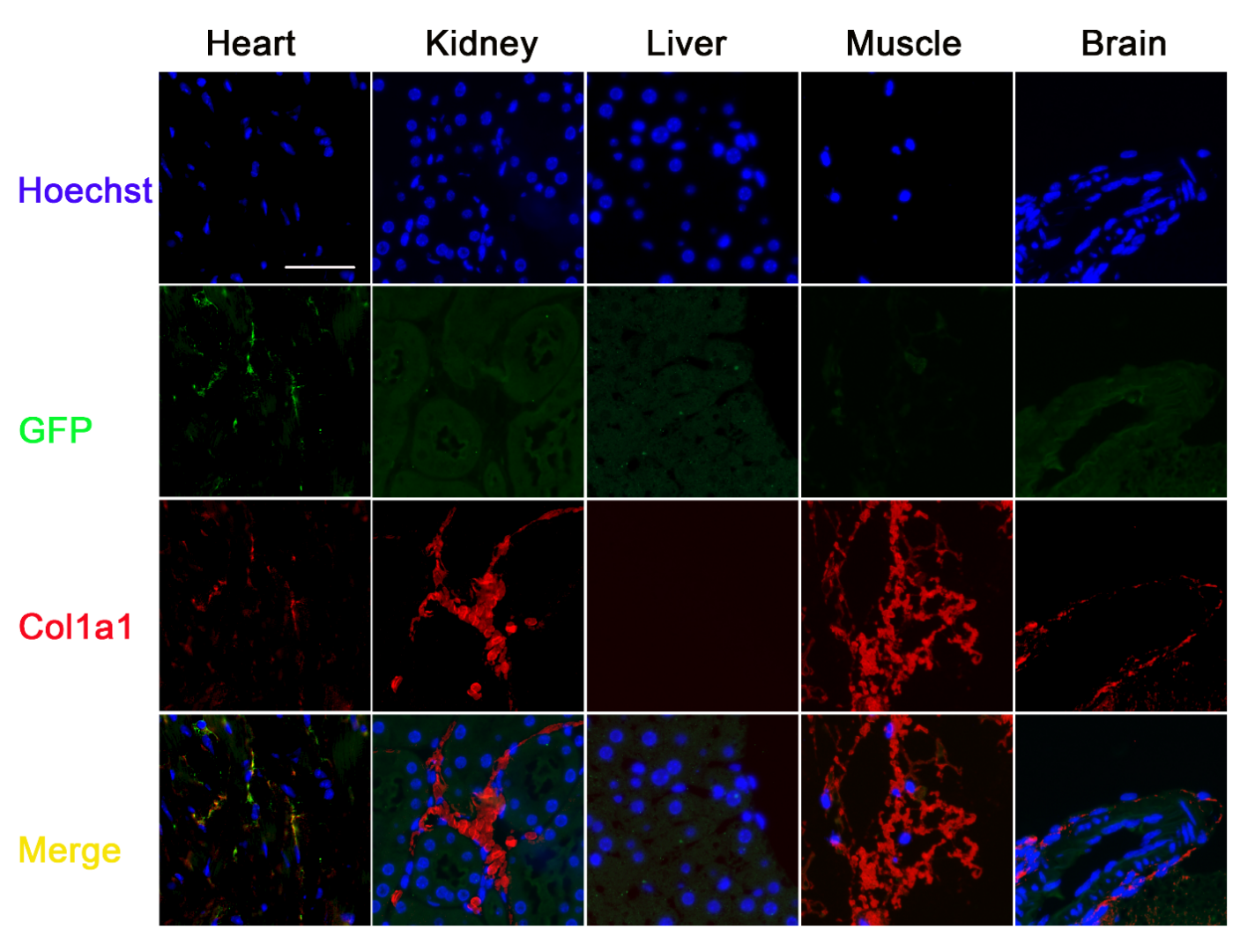
**

(a) Immunofluorescence staining of GFP (green) and fibroblasts (Col1α, red) in the kidney, the liver, the muscle, the brain, and the heart from rAAV-FSP1-GFP treated mice. Scale bar, 100 µm.

**Figure. S15.**

**Representative images of immunofluorescence staining in primary neonatal rat cardiac cells.**

**a**

**
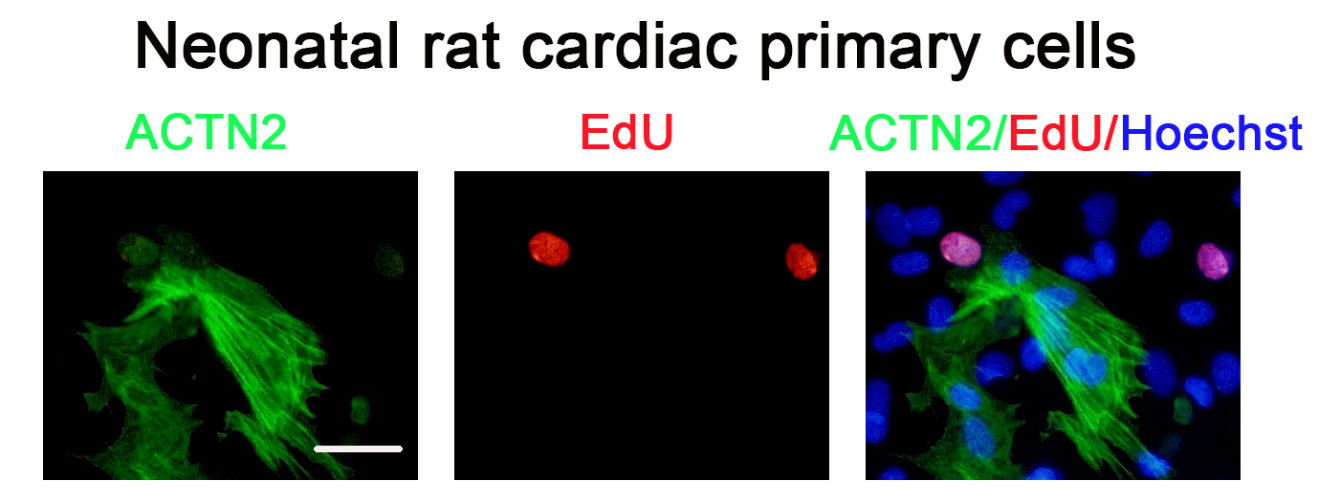
**

(a) Immunofluorescence staining of EdU (red) and CMs (ACTN2, green) in primary neonatal rat cardiac cells. Scale bar, 100 µm.

**Table S1. Clinical characteristics of individuals in the first cohort.**

| **Control** | **Gender** | **Age (years)** | **LVEF** | **Past CVD history** | **Patient** | **Gender** | **Age (years)** | **LVEF (%)** | **Diagnosis** |
| --- | --- | --- | --- | --- | --- | --- | --- | --- | --- |
| 1 | Male | 43 | >50% | No | 1 | Male | 42 | 12 | DCM |
| 2 | Male | 50 | >50% | No | 2 | Male | 68 | 18 | DCM |
| 3 | Male | 46 | >50% | No | 3 | Male | 61 | 26 | ICM |
| 4 | Male | 33 | >50% | No | 4 | Male | 42 | 24 | DCM |
| 5 | Male | 48 | >50% | No | 5 | Male | 60 | 24 | DCM |
| 6 | Male | 36 | >50% | No | 6 | Male | 60 | 22 | DCM |
|  |  |  |  |  | 7 | Male | 66 | 22 | ICM |
|  |  |  |  |  | 8 | Male | 59 | 18 | ICM |
|  |  |  |  |  | 9 | Male | 50 | 22 | ICM |
|  |  |  |  |  | 10 | Male | 52 | 23 | ICM |

LVEF, left ventricular ejection fraction; CVD, cardiovascular disease; DCM, dilated cardiomyopathy; ICM, ischemic cardiomyopathy.

**Table S2. Baseline characteristics of the second cohort.**

| **Variable** | **Control group (n = 32)** | **HF group (n = 32)** | **P-value** |
| --- | --- | --- | --- |
| Male, n (%) | 17 (53%) | 25 (68%) | 0.06 |
| Age (years) | 56 ± 2 | 57 ± 2 | 0.81 |
| Diabetes mellitus, n (%) | 4 (13%) | 3 (9%) | 1 |
| NYHA class |  |  |  |
| II/III/IV | - | 6/15/11 | - |
| LVEF (%) | 68 ± 1 | 27 ± 1 | <0.0001 |
| NT-proBNP (ng/L) | 85.2 ± 8.6 | 8375 ± 1566 | <0.0001 |
| TC (mmol/L) | 4.1 ± 0.1 | 3.8 ± 0.2 | 0.21 |
| Medications, n (%) |  |  |  |
| ACEI or ARB | 11 (34%) | 26 (81%) | <0.001 |
| Diuretic | 1 (3%) | 26 (81%) | <0.0001 |
| Beta blocker | 9 (28%) | 15 (47%) | 0.07 |
| Digoxin | - | 18 (56%) | - |
| MRA | 1 (3%) | 24 (75%) | <0.0001 |

HF, heart failure; NYHA, New York Heart Association; LVEF, left-ventricular ejection fraction; NT-proBNP, N-terminal pro-brain natriuretic peptide; TC, total cholesterol; ACEI, angiotensin converting enzyme inhibitor; ARB, angiotensin II receptor blockade; MRA, mineralocorticoid receptor antagonist; P values represent comparison between HF and control population.

**Table S3. Echocardiographic characteristics of TAC mice at multiple time points.**

|  | Sham | TAC-3d | TAC-7d | TAC-14d | TAC-28d | TAC-70d |
| --- | --- | --- | --- | --- | --- | --- |
| HR (b.p.m.) | 438 ± 19 | 441 ± 12 | 479 ± 13 | 470 ± 18 | 464 ± 19 | 476 ± 8 |
| LVPW, d (mm) | 0.73 ± 0.03 | 0.82 ± 0.04 | 0.76 ± 0.02 | 0.83 ± 0.03* | 0.85 ± 0.04* | 1.07 ± 0.04* |
| LVPW, s (mm) | 1.03 ± 0.03 | 1.13 ± 0.05 | 1.04 ± 0.05 | 1.13 ± 0.04 | 1.25 ± 0.04* | 1.40 ± 0.04* |
| LVAW, d (mm) | 0.89 ± 0.03 | 0.87 ± 0.03 | 0.86 ± 0.04 | 0.99 ± 0.03* | 1.00 ± 0.04* | 1.18 ± 0.06* |
| LVAW, s (mm) | 1.28 ± 0.04 | 1.21 ± 0.04 | 1.24 ± 0.05 | 1.44 ± 0.02* | 1.52 ± 0.03* | 1.67 ± 0.08* |
| LVID, d (mm) | 3.71 ± 0.07 | 3.62 ± 0.13 | 3.71 ± 0.04 | 3.67 ± 0.08 | 3.93 ± 0.08 | 3.82 ± 0.10 |
| LVID, s (mm) | 2.40 ± 0.08 | 2.40 ± 0.07 | 2.60 ± 0.06 | 2.60 ± 0.07 | 2.76 ± 0.07* | 3.00 ± 0.09* |

Values represent mean + SEM; Sham, n = 16; TAC-3d, n = 15; TAC-7d, n = 15; TAC-14d, n = 17; TAC-28d, n = 14; TAC-70d, n = 13; HR, heart rate; LVPW, d, LV posterior wall thickness at diastole; LVPW, s, LV posterior wall thickness at systole; LVAW, d, LV anterior wall thickness at diastole; LVAW, s, LV anterior wall thickness at systole; LVID, d LV internal diameter at diastole; LVID, s, LV internal diameter at systole; d, day. *P <0.05 vs. Sham.

**Table S4. Transcription factors binding to Ago2 promoter predicted by JASPAR among human, rat and mouse.**

| Transcription factors | Predicted site sequence |
| --- | --- |
| ARID3A | ATCAAA |
| ELF1 | AATAAAGGAAATG |
| ESRRB | TGCCAAAGGGCA |
| FOXI1 | TTTTGCTTGTTT |
| GFI1 | GCAATCAGAG |
| KLF4 | GGGGCGGTGC |
| KLF5 | ACCTCACCCC |
| MZF1_1-4 | GGATGGGTGA |
| NR2C2 | AAATGTGAAAGGCCG |
| PRRX2 | AATTA |
| SPI1 | AGAAAGGAGAAGCCC |
| STAT1 | TTTCAAAGAAA |
| STAT5 | CCTTCCCGGTA |
| ZFP423 | GGGCCTTAAGGGGGC |
| ZFX | CACGTCCGGGCCTT |

**Table S5. Upregulated genes by Ago2 mediating RIP-seq in H9c2 cell line.**

| **Gene symbol** | **Ago2-miR320** | **Ago2-MR** | **Fold change (miR320 vs MR)** |
| --- | --- | --- | --- |
| Filip1l | 132.0991945 | 48.69068298 | 2.713028169 |
| Rbm25l1 | 325.3189118 | 158.2447197 | 2.055796316 |
| Nexn | 220.8225341 | 79.12235984 | 2.790899242 |
| Plekhm3 | 48.30492933 | 9.129503058 | 5.291079812 |
| Rundc1 | 63.09215259 | 23.33095226 | 2.704225352 |
| Hnrnpa3 | 157.7303815 | 77.09358138 | 2.04595997 |
| Cep164 | 186.3190131 | 88.25186289 | 2.111219038 |
| Lmod3 | 462.3471807 | 227.2231872 | 2.034771127 |
| Bahcc1 | 67.03541213 | 24.34534149 | 2.753521127 |
| AABR07043748.1 | 170.545975 | 77.09358138 | 2.212194218 |
| Nynrin | 71.96448655 | 26.37411995 | 2.728602384 |
| Myh9l1 | 275.0423527 | 35.503623 | 7.746881288 |
| AABR07028970.2 | 13.80140838 | 0 |  |
| Socs7 | 44.36166979 | 12.17267074 | 3.644366197 |
| Prr12 | 204.063681 | 88.25186289 | 1.209320799 |

**Table S6. Upregulated genes by Ago2 mediating RIP-seq in NRCFs.**

| **Gene symbol** | **Ago2-miR320** | **Ago2-MR** | **Fold change (miR320 vs MR)** |
| --- | --- | --- | --- |
| Cd82 | 21.21320344 | 2.828427125 | 7.5 |
| Fhl1 | 12.02081528 | 0 |  |
| Col6a1 | 18.38477631 | 2.828427125 | 6.5 |
| Baz1b | 21.21320344 | 2.828427125 | 7.5 |
| Pdgfra | 53.74011537 | 16.97056275 | 3.166666667 |
| Vmp1 | 15.55634919 | 0 |  |
| Ifitm1 | 21.92031022 | 4.242640687 | 5.166666667 |
| Rpn2 | 43.84062043 | 12.72792206 | 3.444444444 |
| Tagln2 | 91.92388155 | 24.04163056 | 3.823529412 |
| Sulf1 | 74.24621202 | 26.87005769 | 2.763157895 |
| Zc3h12a | 12.02081528 | 0 |  |
| Hdac1 | 12.02081528 | 0 |  |
| Map7d1 | 21.21320344 | 4.242640687 | 5 |
| Rpl29 | 20.50609665 | 0 |  |
| Serp1 | 20.50609665 | 2.828427125 | 7.25 |
| Sec61a1 | 24.04163056 | 4.242640687 | 5.666666667 |
| Psmd13 | 11.3137085 | 0 |  |
| Fbln1 | 10.60660172 | 0 |  |
| Il1r1 | 11.3137085 | 0 |  |
| Clu | 26.1629509 | 4.242640687 | 6.166666667 |
| Atp5a1 | 23.33452378 | 4.242640687 | 5.5 |
| Tpm3 | 21.92031022 | 4.242640687 | 5.166666667 |
| Anxa1 | 35.35533906 | 9.899494937 | 3.571428571 |
| Golga7 | 11.3137085 | 0 |  |
| Serpinb6 | 10.60660172 | 0 |  |
| Picalm | 23.33452378 | 4.242640687 | 5.5 |
| Smad7 | 16.97056275 | 0 |  |
| St13 | 16.97056275 | 1.414213562 | 12 |
| Nfkb2 | 12.72792206 | 0 |  |
| Ganab | 20.50609665 | 2.828427125 | 7.25 |
| Ssr2 | 12.72792206 | 0 |  |
| Dapk3 | 14.8492424 | 1.414213562 | 10.5 |
| Cfl1 | 56.56854249 | 16.97056275 | 3.333333333 |
| Nucb1 | 91.21677477 | 32.52691193 | 2.804347826 |
| Aco2 | 10.60660172 | 0 |  |
| Pcolce | 66.46803743 | 24.04163056 | 2.764705882 |
| Ppp1r15b | 15.55634919 | 1.414213562 | 11 |
| Ftl1 | 53.74011537 | 4.242640687 | 12.66666667 |
| Larp1 | 10.60660172 | 0 |  |
| Actb | 142.8355698 | 60.81118318 | 2.348837209 |
| Csnk1d | 21.92031022 | 1.414213562 | 15.5 |
| AABR07000398.1 | 358.5031381 | 120.2081528 | 2.982352941 |
| Ak3 | 17.67766953 | 0 |  |
| G6pd | 14.8492424 | 0 |  |

**Table S7. The sequences designed for the expressions of miR-random, miR-320 and miR-320-TUD.**

|  | Sequences (5’ 3’) |
| --- | --- |
| MiR-random | GATCCTTTGTACTACACAAAAGTACTGTTCAAGAGACAGTACTTTTGTGTAGTACAAACCGC |
| MiR-320 | AGCTTTCGCCCTCTCAACCCAGCTTTT TTCAAGAGAAAAAGCTGGGTTGAGAGGGCGACCGC |
| MiR-320-TUD | GACGGCGCTAGGATCATCAACTCGCCCTCTCAAATCTCCCAGCTTTTCAAGTATTCTGGTCACAG  AATACAACTCGCCCTCTCAAATCTCCCAGCTTTTCAAGATGATCCTAGCGCCGTCTTTTTT |

**Table S8. The sequences of primers for mRNA detection.**

| Species | mRNA | Forward (5’-3’) | Reverse (5’-3’) |
| --- | --- | --- | --- |
| Rattus norvegicus | ANP | CGGTACCGAAGATAACAGCC | TGGTGCTGAAGTTTATTCGGATTTA |
|  | β-MHC | TGGCACCGTGGACTACAATA | TACAGGTGCATCAGCTCCAG |
|  | Col1a1 | CCCAGCGGTGGTTATGACTT | CGGCCACCATCTTGAGACTT |
|  | α-SMA | ACCATCGGGAATGAACGCTT | CTGTCAGCAATGCCTGGGTA |
|  | Myh9 | GCCATACAATAAATACCGCTTCC | CTGGTCAGTGTTCCGTTCCTTC |
|  | PLEKHM3 | AGCATATCTGTTTTAGGCAATCTG | TGATGACCAAGTCCTGGAGAGT |
|  | SOCS7 | CAAGAGTCACGTCTGCCAGT | TCAACTGCAGTACCACGTCC |
|  | NEXN  PDGFRa  IFITM1  FHL1  SMAD7 | CCTGAGGACCTGGACAGAGA  CTCACTTTTTCCTCCGGGCT  CGGACCAAGCCTGTATCCTC  CTGGGCTTGAGAGAAGACGG  CGACGAAGAGAGTCTCGGAGGA | TACCATGTTCCGCCTTGCTT  TGTGAGGAGACAGCTGAGGA  TGTGGTGGTTGTCGCAGAAT  CAAAGCACTTCAGGCAGCAG  GCCCCGACCCGACAGT |
| Mus musculus | ANP | AGGCAGTCGATTCTGCTTGA | CGTGATAGATGAAGGCAGGAAG |
|  | BNP | TAGCCAGTCTCCAGAGCAATTC | TTGGTCCTTCAAGAGCTGTCTC |
|  | β-MHC | AGCCTCAGCAGAGGAGTACA | GGCTGAGCCTTGGATTCTCA |
|  | Ago2 | TCCATGAGGTACACCCCTGT | GATGGAAGCCAAACCACACT |
|  | Col1a1 | CCGAGGTATGCTTGATCT | GACAGTCCAGTTCTTCATTG |
|  | Fibronectin | GTGTCTATGCTCTCAAGGA | CTAATAGTGATGGTGGTCTCT |

**References**

1. Wu J. et al. Effects of heart rate and anesthetic timing on high-resolution echocardiographic assessment under isoflurane anesthesia in mice. *Journal of ultrasound in medicine : official journal of the American Institute of Ultrasound in Medicine*. 2010;29:1771-8.

2. Han F. et al. MiR-217 mediates the protective effects of the dopamine D2 receptor on fibrosis in human renal proximal tubule cells. *Hypertension (Dallas, Tex : 1979)*. 2015;65:1118-25.

3. Nie J. et al. Ranolazine prevents pressure overload-induced cardiac hypertrophy and heart failure by restoring aberrant Na(+) and Ca(2+) handling. *Journal of cellular physiology*. 2019;234:11587-11601.

4. Sassi Y. et al. Cardiac myocyte miR-29 promotes pathological remodeling of the heart by activating Wnt signaling. *Nature communications*. 2017;8:1614.

5. Thum T. et al. MicroRNA-21 contributes to myocardial disease by stimulating MAP kinase signalling in fibroblasts. *Nature*. 2008;456:980-4.

6. Li H. et al. MicroRNA-21 Lowers Blood Pressure in Spontaneous Hypertensive Rats by Upregulating Mitochondrial Translation. *Circulation*. 2016;134:734-51.
